# SOFB is a comprehensive ensemble deep learning approach for elucidating and characterizing protein-nucleic-acid-binding residues

**DOI:** 10.1038/s42003-024-06332-0

**Published:** 2024-06-03

**Authors:** Bin Zhang, Zilong Hou, Yuning Yang, Ka-chun Wong, Haoran Zhu, Xiangtao Li

**Affiliations:** 1 School of Artificial Intelligence, Jilin University, Changchun, China; 2 Donnelly Centre for Cellular and Biomolecular Research, University of Toronto, Toronto, Canada; 3 Department of Computer Science, City University of Hong Kong, Hong Kong, Hong Kong SAR

**Keywords:** Data mining, Sequence annotation

## Abstract

Proteins and nucleic-acids are essential components of living organisms that interact in critical cellular processes. Accurate prediction of nucleic acid-binding residues in proteins can contribute to a better understanding of protein function. However, the discrepancy between protein sequence information and obtained structural and functional data renders most current computational models ineffective. Therefore, it is vital to design computational models based on protein sequence information to identify nucleic acid binding sites in proteins. Here, we implement an ensemble deep learning model-based nucleic-acid-binding residues on proteins identification method, called SOFB, which characterizes protein sequences by learning the semantics of biological dynamics contexts, and then develop an ensemble deep learning-based sequence network to learn feature representation and classification by explicitly modeling dynamic semantic information. Among them, the language learning model, which is constructed from natural language to biological language, captures the underlying relationships of protein sequences, and the ensemble deep learning-based sequence network consisting of different convolutional layers together with Bi-LSTM refines various features for optimal performance. Meanwhile, to address the imbalanced issue, we adopt ensemble learning to train multiple models and then incorporate them. Our experimental results on several DNA/RNA nucleic-acid-binding residue datasets demonstrate that our proposed model outperforms other state-of-the-art methods. In addition, we conduct an interpretability analysis of the identified nucleic acid binding residue sequences based on the attention weights of the language learning model, revealing novel insights into the dynamic semantic information that supports the identified nucleic acid binding residues. SOFB is available at https://github.com/Encryptional/SOFB and https://figshare.com/articles/online_resource/SOFB_figshare_rar/25499452.

## Abstract (web summary)

We implement SOFB, an ensemble deep learning model-based nucleic-acid-binding residues on proteins identification method to characterize protein sequences by learning the semantics of biological dynamics contexts.

## Introduction

Protein-nucleic acid interactions are part of many fundamental cellular functions, such as DNA transcription, replication, protein synthesis, regulation of gene expression, post-transcriptional modifications, and cellular regulation^1,2^. The identification of specific binding sites in proteins is particularly crucial for understanding the function of protein molecules and for designing new therapeutic compounds to modulate protein functions in diseases^3^. To accelerate the characterization of protein-DNA and protein-RNA interactions, computational methods have been proposed to detect DNA or RNA-binding residues in protein sequences using the sequence information or structural information of the protein^4^.

Generally, the algorithms can be divided into two broad categories: (1) models that rely on sequence information and (2) models that use structural information. The former approach investigates the protein by sequence information and leverages a model to target the nucleic-acid-binding residues: for instance, iDRNA-ITF constructs an induction and transfer framework to enable the representation of residues including functional description^5^. In addition, other sequence-based methods such as TargetDNA^6^, DRNApred^1^, TargetS^7^, SVMnuc^8^, RNABindRplus^9^, have been widely employed. The advantage of the sequence-based approach is that all proteins with known sequences can be examined, which is practical^10^. The second approach, introduces structural information on top of the former information to make joint prediction: for instance, Xai et al. developed GraphBind that uses hierarchical graph neural networks to process spatial features for classification^2^. On this basis, despite the proliferation of protein sequence data driven by advancements in second-generation sequencing technology, the information gap between protein-nucleic-acid complexes and their corresponding structural and functional data in the Protein Data Bank (PDB)^11,12^ remains large, posing a challenge for the development of effective computational methods for proteins lacking structural information. As such, the urgency to develop a high-throughput, precise, and resilient method that can predict nucleic-acid-binding residues based purely on sequence information cannot be understated.

On the other hand, the current sequence-based protein feature representations are still limited to biological features, which typically, only extract a portion of the interactions between residues and do not provide a complete protein sequence map. As we know, protein sequences are like human language in both representation and semantics, and inspired by this, many researchers have solved problems by considering sequences as languages, and then use approaches in that field. However, these studies have only utilized static language models, neglecting the global-based dynamic semantic information of protein sequences and, thus, inadequately representing the deeper information of sequences. To address the shortcomings of these methods, the Transformer-based language model approach has been successfully extended to the protein domain. There have been studies that have utilized that to encode biological sequences^13,14^. Specifically, Wang et al. proposed SMFM^15^, which utilizes a fine-tuned BERT^16^ model to comprehensively identify and analyze enhancers from regulatory DNA sequences. Zhu et al. proposed HDRNet^17^, which employed dynamic coding to predict dynamic RBP binding events across diverse cellular conditions, providing different insights into the pathological mechanisms underlying RNA-RBP interactions from various perspectives. Like the static approach, the language model treats the entire protein sequence as a sentence and its constituent amino acids as individual words^18,19^. Then, by self-supervised pre-training of a large-scale unlabeled text corpus, efficient representations containing global sequence information with including deeper semantic information can be generated.

Here, we propose the SOFB, an ensemble deep learning-based **S**equence network that utilizes **O**nly sequence information to **F**ind nucleic-acid-**B**inding residues. Specifically, for a given protein sequence, we aim to identify whether each amino acid in the protein sequence is a nucleic acid binding residue. To capture a more accurate and detailed representation of amino acids within proteins, multi-source biological features and dynamic residue language embedding model are employed to represent the protein sequences, where dynamic residue language embedding model can maximize the characterization power of the dynamic semantic information of protein sequences. After that, an ensemble deep learning-based sequence network, consisting of a diverse range of convolutional layers in conjunction with Bi-directional Long Short-Term Memory (Bi-LSTM), is designed to process and refine the various features for optimal performance. To validate the effectiveness and good performance of SOFB, we conducted several experiments based on several DNA/RNA nucleic-acid-binding residue datasets, demonstrating that SOFB outperforms currently available methods. In addition, to elucidate the underlying reasons for the identified nucleic acid binding residues, we conducted an interpretability analysis based on the attention weights of the language learning model, revealing novel insights into the dynamic semantic information supporting the identified nucleic acid binding residues.

## Results

### Data sources

To evaluate the effectiveness of SOFB and establish a valid comparison with alternative methodologies, we collected two benchmark datasets of nucleic-acid-binding proteins from the reference^2^. Each of these datasets comprises a training set and a test set, termed as DNA-573_train, DNA-129_test, RNA-495_train, and RNA-117_test. Specifically, the nucleic-acid-proteins in these datasets were sourced from the BioLip database^20^, which stores information on biological ligand-protein interactions. BioLip contains multiple types of DNA-RNA-protein complexes in which many nucleic-acid-binding sites are labeled at binding residues defined as amino acids with a minimum atomic distance of less than 0.5 Å between the target residue and the nucleic-acid molecule, plus the sum of the Van der Waal’s radius of the two nearest atoms. To facilitate subsequent training, protein complexes containing only DNA or RNA in BioLip were selected. Ultimately, 9574 DNA-protein chains and 7693 RNA-protein chains were chosen among the protein chains containing nucleic-acid-protein binding sites. In addition, we increased the number of positive samples by amplifying the annotation with similar protein sequences, which effectively reduce the effect of imbalance between positive and negative samples.

In particular, we first identify the analogous protein chains by calculating sequence identity and structural similarity using bl2seq (E-value <0.001) and TM-align^21^ between pairs of protein chains. Then, we performed clustering on protein chains exhibiting sequence identity >0.8 and TM score >0.5. Subsequently, annotations from the protein chains within each cluster were transferred to the chain with the largest number of residues. Moreover, to refine the training sets, we further pruned protein chains with sequence identity lower than 30% using the CD-HIT method^22^. As a result, the number of DNA and RNA binding residues were increased by 30.7% and 24.3%, respectively.

In particular, the DNA training and test sets consist of 573 and 129 protein chains, respectively, while the RNA training and test sets encompass 495 and 117 protein chains, respectively. The DNA training set contains 14,479 binding residues and 145,404 non-binding residues, whereas the RNA training set comprises 14,609 binding residues and 122,290 non-binding residues. To demonstrate the predictive performance and ensure a rigorous assessment of the model’s capabilities, we partitioned the initial training sets into a training set (80%) and a validation set (20%). Subsequently, we processed the test sets, which contained 2,240 and 35,275 binding and non-binding residues for DNA, and 2,031 and 35,314 binding and non-binding residues for RNA, respectively, to perform an independent test. (See Supplementary Table 1, Supplementary Note 1)

### The overview of SOFB

**Fig. 1:**
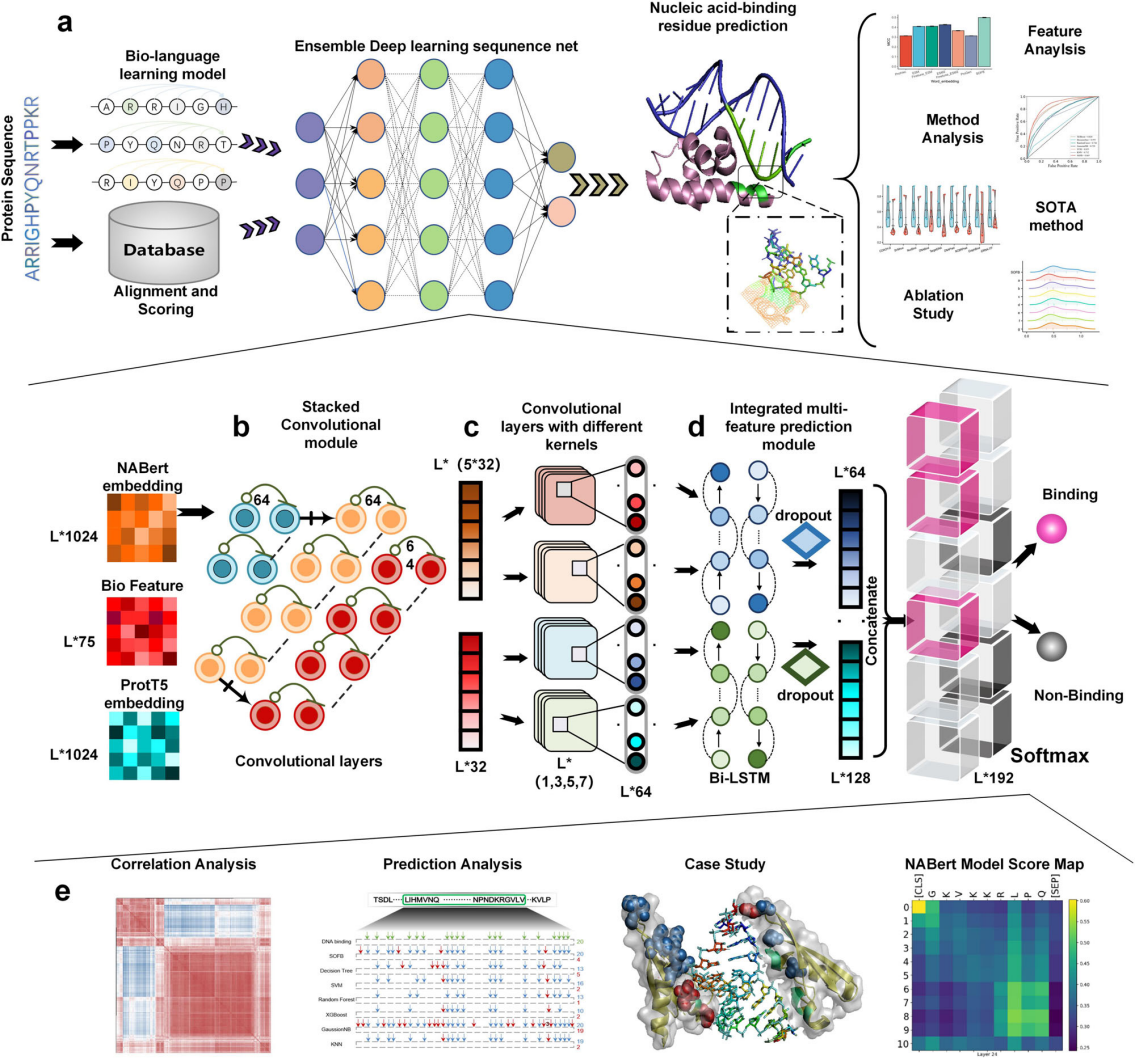
The overview of SOFB. **a** A comprehensive framework for SOFB, an end-to-end architecture for identifying nucleic-acid-binding residues. Concretely, sequences are fed into the language learning model for fine-tuning. Firstly, the representation of the hidden layer (NABert embedding) is fed into the stacked convolutional module within SOFB, and the states of the features of several convolutional layers are also recorded as one of the features to be used subsequently. Moreover, the extracted features are combined with the biological features and passed through the convolutional layers with different convolutional kernel sizes within SOFB. Afterwards, the learned features are integrated into the Bi-LSTM together with the ProtT5 embedding learned in large-scale sequences. Finally, the prediction is performed using the fully connected layer prediction module. **b** The settings of the stacked convolutional module within the ensemble deep learning-based sequence network. **c** Details of the convolutional layers with different convolutional kernel sizes within the ensemble deep learning-based sequence network. **d** Location and settings of the integrated multi-feature prediction module in SOFB. **e** Further experimental analysis of specific experiments and algorithms, including correlation analysis, prediction analysis, case study and the language model interpretability analysis, etc. Source data are provided with this paper.

The overall network architecture of the SOFB is illustrated in Fig. 1, showing a sequence-driven nucleic-acid-binding residue prediction model using an ensemble deep learning-based sequence network. In general, it involves two parts: the protein language learning enhancement and feature construction, the ensemble deep learning-based sequence network.

Given a protein sequence, our method first uses the strategies to construct various features. Then, these features can be recursively processed by the ensemble deep learning-based sequence network, which contains three modules (the stacked convolutional module, convolutional layers with different convolutional kernels sizes and the integrated multi-feature prediction module) under ensemble learning. In the first step, NABert embedding obtained from our language learning model is fed into the stacked convolutional module containing a single convolutional layer at the front, 13 normal blocks and 3 extra connected blocks to reduce redundant information and to obtain precise global dynamic protein semantic information. Both types of blocks have two convolutional layers. In addition, the state change records are created to record the features before they enter the stacked module, after they leave the module and each time they pass through the extra connected blocks, describing the changes of the features. Then, the 75-dimensional features are united with the features leaving the stacked convolutional module to yield global and particular ranges of local information of proteins by using convolutional layers with convolutional kernel size of 1, 3, 5 and 7, respectively. Then, the integrated multi-feature prediction module is composed of the Bi-LSTM containing 64 units with fully connected layers. To get a better performance of SOFB on unknown protein sequences, we feed this module with the ProtT5 embedding combined with the output of the previous module and the NABert embedding change records, to achieve complementarity between all features. This results in better identification of DNA and RNA-binding residues in the sequence.

### SOFB can provide a more efficient scheme for characterizing nucleotide sequences

To demonstrate the powerful superiority of dynamic language models and their ability to better learn the relationships between amino acids in protein sequences, we compared our model with several other protein word embedding methods, including ProtVec^23^, ESM (esm1_t34_670M_UR100)^24^, ESM2 (esm2_t12_35M_UR50D)^25^ and ProGen^26^. Specifically, ESM, ESM2 and ProGen are protein language models based on the Transformer model, which can provide dynamic contextual embedding for proteins. Moreover, for the ESM and ESM2, we conducted fine-tuning on the training set with 1 epoch and learning rate 0.00005 to enhance its ability to generate word embeddings better suited for the current task. For a fair comparison, we replaced the embeddings generated by other word embedding methods with the NABert embedding learned by the NABert language model in our model.

**Fig. 2:**
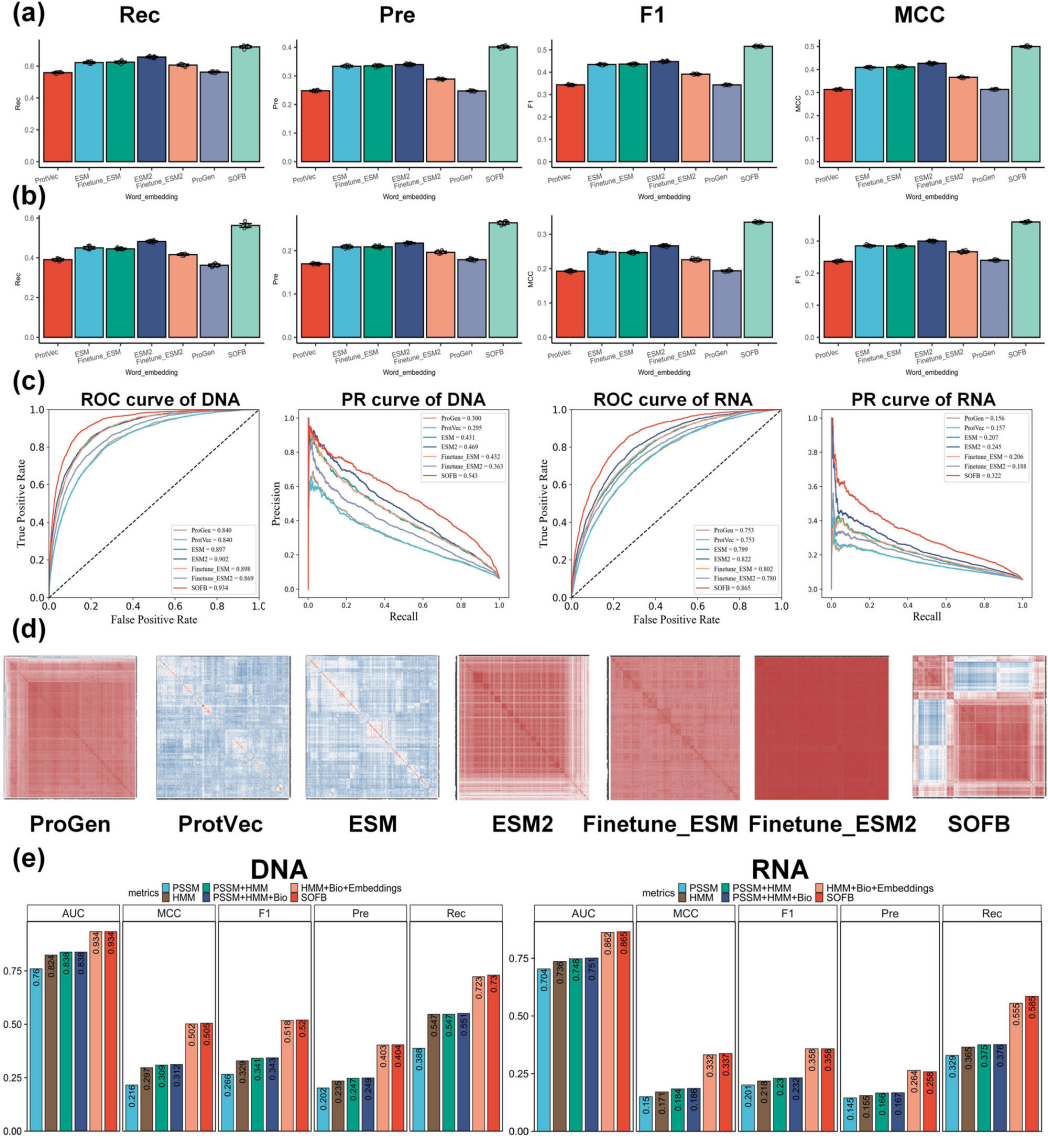
SOFB utilizes a feature combination strategy that works better than other protein characterization approaches. **a** The average Recall (Rec), Precision (Pre), F1, MCC of ten runs of six dynamic contextual embeddings (ProtVec, ESM, Finetune_ESM, ESM2, Finetune_ESM2, ProGen) on DNA-binding test set; **b** shows the average Recall (Rec), Precision (Pre), F1, MCC of ten runs of six dynamic contextual embeddings (ProtVec, ESM, Finetune_ESM, ESM2, Finetune_ESM2, ProGen) on RNA-binding test set; (**c**) provides ROC curves with AUC values, PR curves with AP values for DNA-binding residue and RNA-binding residue predictions, respectively, where SOFB performs best by both metrics. **d** The heat maps of correlation analysis of six dynamic contextual embeddings and NABert embedding on DNA-binding test set; **e** shows the performance of SOFB on different combinations of features on DNA-binding and RNA-binding test sets, where SOFB is PSSM + HMM + Bio + Dynamic residue language embeddings. Source data are provided with this paper.

The experimental results are summarized in Fig. 2 and the Supplementary Fig. 1 of the Supplementary Note 2, where for the DNA-binding test set, the results of Rec, Pre, F1 and MCC metrics are presented in Fig. 2a. As depicted in this figure, the average Rec, Pre, F1 and MCC of dynamic language embeddings in our SOFB are 73.0%, 40.4%, 43.5%, and 40.9%, respectively. It is worth noting that F1 is 0.072 to 0.176 higher than the value of the other methods, including ESM, ProGen, ESM2, Finetune_ESM, Finetune_ESM2 and ProtVec. We also provide the ROC curves of all methods with PR curves in Fig. 2c. We observe that the area under the ROC curve of SOFB on the DNA-binding test set is 93.4%, which is much better than the other methods. The reason is the adaptability of the language learning model used by the multi-feature of SOFB on the nucleic-acid-binding residue task. It enables better learning compared to other dynamic contextual embeddings. For the RNA-binding test set, the results are summarized in Fig. 2b. Compared to the other protein word embedding approaches, SOFB fared the best in finding RNA-binding residues in protein sequences, as measured by all metrics. SOFB increased F1 and MCC metrics by 6.2% and 7.0% over the second-best embedding method, respectively. From the ROC and PR curves in Fig. 2c, we see that the area under the ROC curve of SOFB on the RNA test set is 86.5%, which is 4.3% greater than the better performing ESM2 and 11.2% greater than the worse performing ProtVec and ProGen.

To explore the learned features further, we computed their correlation matrix, as visualized in Fig. 2d for DNA-binding task (see Supplementary Fig. 2, Supplementary Note 3). We observe that SOFB performs better than all other approaches in categorizing amino acids and dividing the amino acids in the sequence into two groups for later identifying nucleic-acid-binding residues. In addition, SOFB uses multiple features for protein representation, including PSSM, HMM, Bio (physicochemical features, PKx and RAA, one-hot), and dynamic language embeddings. To demonstrate the impact of the different feature combinations on SOFB, we evaluated SOFB with the following six feature combinations: (i) PSSM, (ii) HMM, (iii) PSSM + HMM, (iv) PSSM + HMM + Bio, (v) HMM + Bio + Embeddings and (vi) PSSM + HMM + Bio + Embeddings (SOFB). Figure 2e depicts the effects of the different feature combinations on the DNA-binding and RNA-binding test sets, respectively. When using individual features for prediction, HMM has a better separation ability compared to PSSM. SOFB worked better in the RNA task when combining HMM and PSSM, which also reflects the complementarity between the features and the differences between the two tasks. In terms of the PSSM feature, we can observe from Fig. 2e that PSSM plays important in the prediction task of amino acid-nucleic acid binding. Finally, combining all the features, including dynamic residue language embeddings, yielded the highest metrics in all of SOFB, indicating that fusing multi-source biological features and natural language processing methods can learn more information about amino acid positions and deep semantic information about protein sequences.

### SOFB has better performance than most machine learning methods

**Fig. 3:**
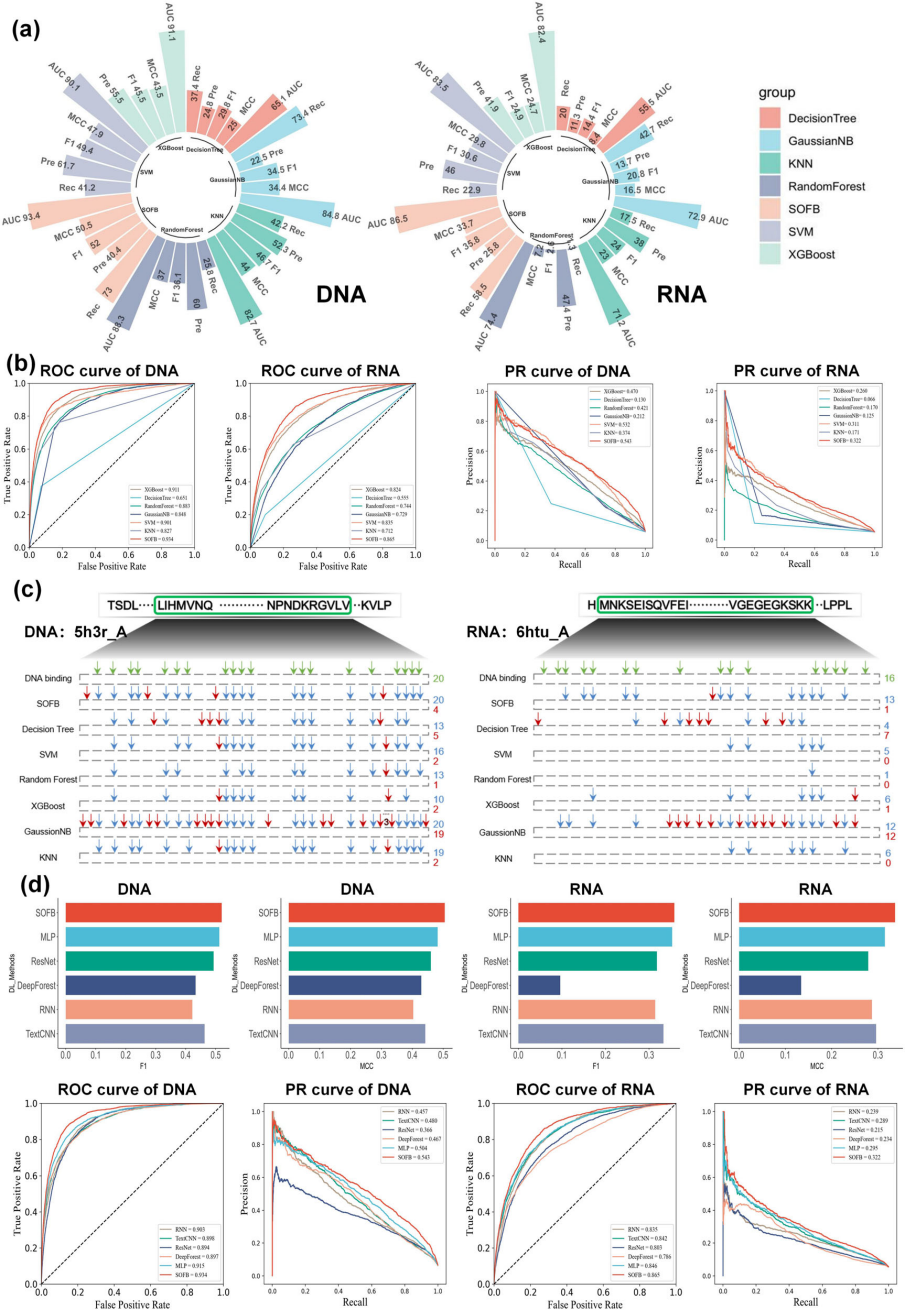
SOFB has a better performance than other machine learning and deep learning methods. **a** The performance comparison of SOFB with six machine learning algorithms on two nucleic-acid-binding test sets; **b** gives the PR curves and ROC curves of machine learning algorithms and SOFB on two tasks, where SOFB’s AUC is higher than the other algorithms; **c** shows the predictions of the different methods on a particular DNA (RNA)-binding protein(the highest MCC),where the green means the nucleic-acid-binding residues, the blue represents the nucleic-acid-binding residues that were successfully predicted and the red means the false positive residues; **d** shows the F1 value vs. MCC value for the different deep learning methods in the DNA-binding residue and RNA-binding residue recognition task, where the result of SOFB is better than all other methods, which also shows the ROC curves and AUROC, PR curves and AP for different deep learning methods in the DNA-binding residue versus RNA-binding residue recognition task. Source data are provided with this paper.

In this section, we compared SOFB with six machine learning algorithms including XGBoost^27^, KNN^28^, GaussianNB^29^, Decision Tree^30^, Random Forest^31^ and SVM^32^. To carry out a fair comparison, we used the same feature representation to measure the performance of these machine learning methods to identify nucleic-acid-binding residues in the two datasets. In addition, to ensure that we do not have any human intervention or bias in parameter tuning, all parameters of different machine learning methods follow the default settings of scikit-learn. As demonstrated in Fig. 3 (a) and (b), compared with the six other machine learning algorithms, SOFB performs better on both the DNA-binding and RNA-binding test sets, and achieves the highest AUC, MCC and F1 values. Moreover, we observe that SOFB improves the AUC from 0.023 to 0.283 on the DNA-binding test set. Further, it is worth noting that SVM outperforms the other machine learning algorithms in both tasks, which may be due to its internal kernel function that can oversee the high-dimensional features well. Our proposed model implies that deep learning is better at learning potential representations of the high-dimensional features to provide superior performance. In addition, we also measure the performance of the different methods by plotting the PR curves based on the prediction, as illustrated in Fig. 3b. We see that the areas under the PR curves of SOFB for the DNA-binding and RNA-binding test sets are 0.543 and 0.322, respectively. These are the best results of all the computational methods, showing that SOFB works well and finds more positive samples.

In addition, to further explore the performance of the various computational methods to identify positive samples, we selected DNA-binding protein sequences and RNA-binding protein sequences for in-depth analysis. The results on the two benchmark tests are displayed in Fig. 3c. For the DNA-binding test set, the sequences contains 20 binding sites. SOFB and GaussianNB both successfully found all binding sites. SVM and KNN found 16 and 19 binding sites, Decision Tree and Random Forest found 13 binding sites, and XGBoost only found 10 binding sites. SOFB actually found more binding sites as it achieved the highest overall index. GaussianNB, on the other hand, predicted 19 pseudo sites, which results in its performance being lower than that of SOFB. For the RNA-binding test set, there are 16 binding sites in the sequence. Decision Tree, SVM, Random Forest, XGBoost and KNN identified 4, 5, 1, 6, and 6 binding sites, respectively. In contrast, SOFB and GaussianNB successfully identified 13 and 12 binding sites, respectively. Notably, GaussianNB predicted 12 incorrect sites, whereas SOFB predicted only one incorrect locus. Consequently, SOFB performs better than GaussianNB. The absence of erroneously predicted loci in SVM, Random Forest and KNN gives them a very high Pre value, yet these approaches overlook a large number of positive sites. Ultimately, their overall metrics are poor. We can conclude that SOFB effectively finds more DNA and RNA-binding residues while reducing the prediction error rate.

### SOFB can provide better performance than the compared deep learning methods

To further demonstrate the superiority of SOFB, we went on to compare it to five deep learning algorithms, including TextCNN, RNN, ResNet, DeepForest and MLP. The experimental results are summarized in Fig. 3d. SOFB outperforms all the deep learning algorithms, and achieved 93.4% and 86.5% of AUC in the DNA-binding and RNA-binding test sets, respectively. In particular, SOFB outperformed TextCNN, RNN, ResNet and DeepForest by 2.3%, 3.0%, 6.2% and 7.9%, respectively in the RNA task. In addition, MCC provided an improvement of 0.041 to 0.203 in the overall results, and F1 provided an improvement of 0.025 over the TextCNN, rated second. Besides, we also compare the simple deep network structure MLP. Although the MLP outperforms all the other deep learning based methods, which we hypothesize is due to the robustness of our features that allow the MLP to fit in well. However, the MLP is still not as good as SOFB. For example, our metrics are all better than MLP in Fig. 3d, and in the ROC plot we find that the AUC of MLP in DNA binding residue recognition reaches 91.5%, which is 1.9% lower than SOFB. In RNA binding residue recognition, the AUC is 84.6%, also 1.9% lower than ours, which reflects the rationality and effectiveness of the structure of our SOFB. We also note that RNN outperforms the rest of the deep learning methods except for SOFB on the DNA-binding residue recognition task, while as stated above, TextCNN achieved the second-best result to SOFB on the RNA-binding residue recognition task, demonstrating the inherent variability in these two identification tasks and the large performance differences between the different models. Besides, we conducted another experiment to investigate the effect of chain interactions on the prediction performance of our SOFB, as illustrated in the Supplementary Table 2, the Supplementary Fig. 3 of the Supplementary Note 4, from which we can observe that although the number of protein chains in the training set had a slight impact on the performance of SOFB, an increase in the number of chains enhanced the interaction between protein chains, thereby further improving the predictive capability of SOFB.

### SOFB can perform better than other nucleic-acid-binding residue identification methods

**Fig. 4:**
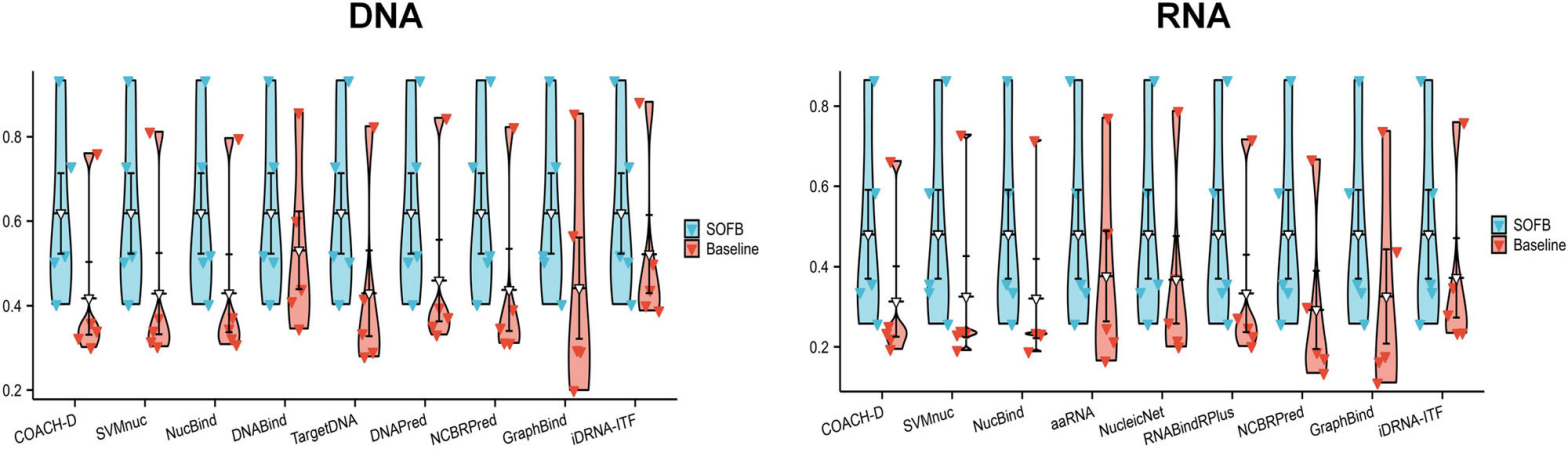
SOFB predicts nucleic-acid-binding residues more accurately than the other state-of-the-art methods. It provides a comparison of SOFB with other state-of-the-art algorithms on the DNA (RNA)-binding test sets, where other algorithm results are reported in Ref. ^5^. The violin plots depicting multiple performance metrics of different baseline methods along with SOFB, where SOFB outperforms all other methods. The triangle markers means Recall (Rec), Precision (Pre), F1 score, Matthews correlation coefficient (MCC) and Area Under the Curve (AUC) (n=5). Source data are provided with this paper.

To verify that the features and network architecture used by SOFB can learn the sequence information of proteins better and effectively identify the nucleic-acid-binding residues, we compared the models with several current state-of-the-art methods for predicting nucleic-acid-binding residues in proteins. For DNA-binding residue predictions, we compared TargetDNA, DNABind^33^, GraphBind, etc. And for RNA-binding residue predictions, we compared aaRNA^34^, RNABindRPlus and iDRNA-ITF, etc. We used Rec, Pre, MCC, F1 and AUROC metrics to measure how well the methods work, as well as the different computational models work. In Fig. 4, the performance of the different state-of-the-art methods and SOFB on the two nucleic-acid-binding test sets, we see that our method SOFB achieves a particularly good performance in both identification tasks. The performance of SOFB was obviously improved, however, only the Pre of NCBRPred was slightly higher than that of SOFB. In addition, we see that SOFB achieves 0.520 (0.358), 0.505 (0.337) and 0.934 (0.865) for F1, MCC, and AUROC on the DNA-binding and RNA-binding test sets, respectively. Notably, the value of AUROC improves by 5.1% on the DNA-binding test set and by 10.5% on the RNA-binding test set compared to the current best performing method, iDRNA-ITF (See Supplementary Table 3, Supplementary Fig. 4 of Supplementary Note 5). The reason could be that the dynamic language embedding of SOFB learning can effectively characterize the relationship between the different amino acids of a protein, and the complementary effect between multiple features allow a clear-cut understanding of the properties of each amino acid, ensuring better identification results. In addition, we also conducted experiments on evaluating the performance of our SOFB on predicting nucleic acid binding residues of proteins across different protein families. And we have validated that there exists particular types of proteins that SOFB performs well, as summarized in the Supplementary Fig. 5 of the Supplementary Note 6.

### Ablation studies on SOFB

To demonstrate the improvement brought by the different modules of SOFB, we performed ablation experiments with testing on the DNA (RNA)-binding test sets. We eliminated different modules within SOFB, including the stacked convolutional module, convolutional layers with different convolutional kernel sizes, the integrated multi-feature prediction module and module that memorize the NABert embedding change records. The different settings are described as four cases: (a) removing the Bi-LSTM of the integrated multi-feature prediction module from SOFB for simple feature union; (b) removing the convolutional layers with different convolutional kernel sizes from SOFB and using only the stacked convolutional module for prediction; (c) removing the stacked convolutional module from SOFB and using only convolutional layers with different convolutional kernel sizes for prediction; (d) removing the Bi-LSTM that processes the feature (*f* ^*state*^) from SOFB to verify its contribution. Experimental results on the DNA-binding and RNA-binding test sets under these different conditions are shown in the Supplementary Fig. 6 of the Supplementary Note 7. We see from these plots that the results for SOFB complete are higher than all other settings. The AUC was lower in the DNA and RNA prediction tasks by 0.2%–2.9% and 0.3%–4.3%, respectively and F1 decreased by 0.8%–7.6% and 0.1%–4.7%, respectively and MCC decreased by 0.8%–7.9% and 0.4%–5.8% respectively. Thus, each module in SOFB contributes to the prediction. Settings (a) verifies that the Bi-LSTM in the integrated multi-feature prediction module can integrate perfectly all the information to improve the prediction results. From settings (b), we understand that the convolution by the different convolutional kernel sizes can obtain different ranges of effective information. The setting (c) of SOFB illustrates that the stacked convolutional layers can extract a large amount of effective information in the features. Finally, the setup in (d) provides valuable evidence that the Bi-LSTM that processes the feature (*f* ^*state*^) plays an active role. In conclusion, the experiments in this section illustrate the rationality and complementarity of the module setup of SOFB.

We also performed ablation experiments for the feature matching, i.e., ablation of matching the relationships between the distinct features and different modules of SOFB. For this purpose, three experiments were set up: (e) replacing the NABert embedding with ProtT5 embedding for prediction; (f) replacing the ProtT5 embedding with the NABert embedding for prediction as well; (g) exchanging the processing modules of the NABert embedding and the ProtT5 embedding for prediction. The results of the three ablation experiments on the DNA-binding and RNA-binding test sets are also shown in the Supplementary Fig. 6 of the Supplementary Note 7. The AUC of all three experiments e, f, and g decreased by 0.3%-2.9% and 0.3%-3.6% on the two test sets, respectively. F1 decreased by 1.1%-5.5% and 0%-3.5%, respectively. MCC decreased by 1.0%–6.2% and 0.3%–4.6%, respectively. The results of the setting e and f show that both features contain particular information. In addition, the setting g shows that the distinct features contain information with specific meaning. The NABert embedding contains information adapted to the nucleic-acid-binding residues datasets, and it is difficult to mine its complex information in Bi-LSTM. Thus, the processing of the stacked convolutional module is needed to get valuable information. And the ProtT5 embedding contains generalized information, while the excessive processing will make the model lose its task-specific nature. Only an appropriate processing of each feature brings out its best effect, and an unsuitable operation will lead to ignoring the effective information. SOFB achieves an excellent match with its features. Furthermore, to explore and compare the predictive capabilities of SOFB, we collected YFK16, YK17 and MW15 test datasets from ref. ^35^ to verify the effectiveness of SOFB. We also selected the YK17 training dataset as our large-scale benchmark dataset to examine the performance of SOFB on a larger number of protein sequences as summarized in the Supplementary Table 4, the Supplementary Table 5 of the Supplementary Note 8. The results show that SOFB achieves the best performance on numerous datasets.

### Case study

To further explore the performance of our proposed methods, we compared SOFB with the second-best method iDRNA-ITF to visualize the nucleic-acid-binding residues in the DNA-binding and RNA-binding test sets. We selected the three protein chains with the highest MCC score in two datasets. For the DNA task, we chose 5h3r_A, 6c31_A, 6enb_A DNA-binding proteins, and for the RNA task, we chose the 6htu_A, 5www_A and 5wzg_A RNA-binding proteins (see Supplementary Fig. 7, Supplementary Fig. 8 of Supplementary Note 9).

**Fig. 5:**
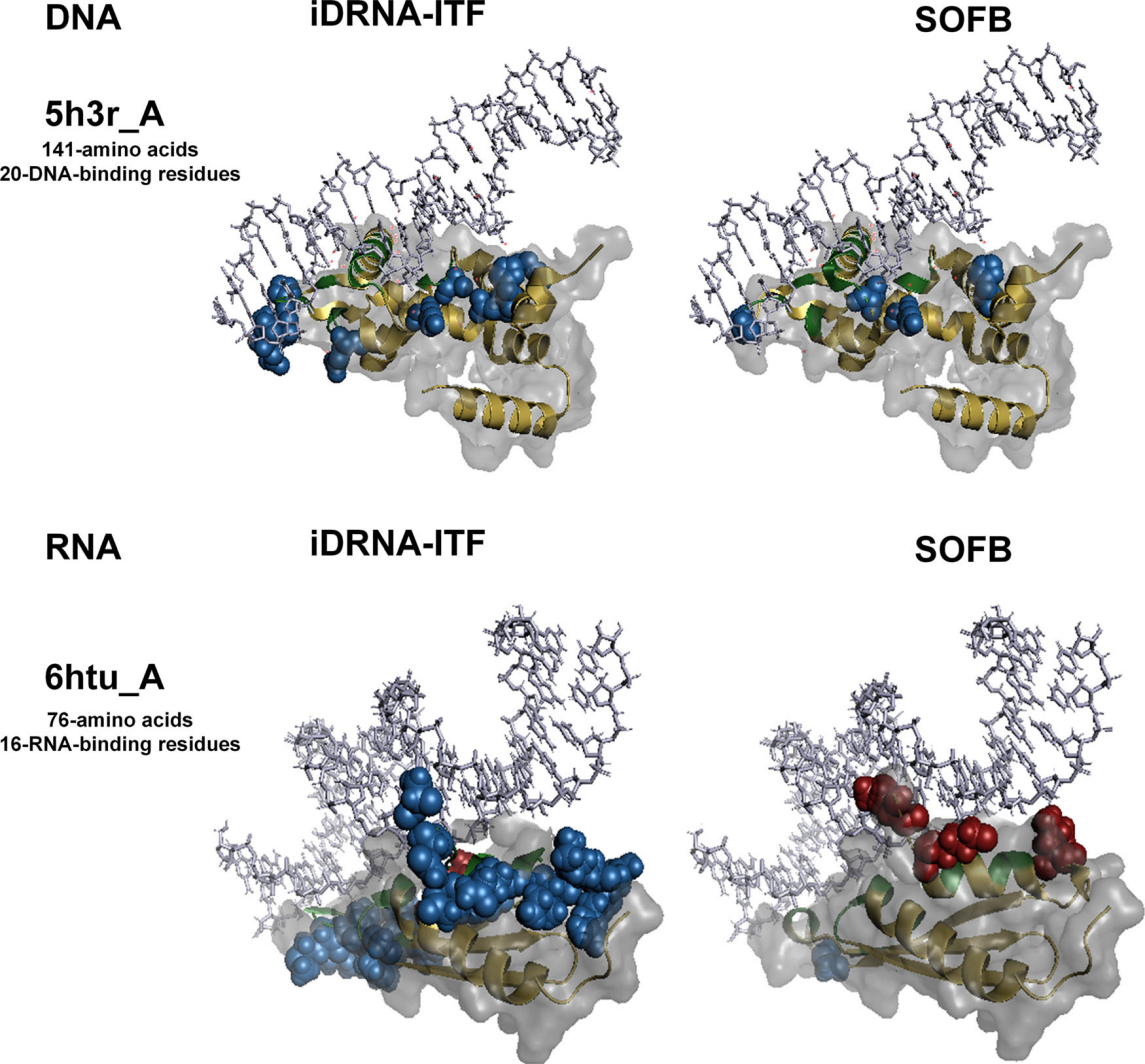
Visualization of two cases predicted by SOFB and the second-best method, iDRNA-ITF. For the protein chain 5h3r_A from DNA-129_Test, the results predicted by iDRNA-ITF (left) and SOFB (right) are shown. For the protein chain 6htu_A from RNA-117_Test, the results predicted by iDRNA-ITF (left) and SOFB (right) are shown, where the red spheres are residues predicted as false negatives, the blue spheres are residues predicted as false positives, the green represents true positive residues, and the yellow are true negative residues. Out of the white protein surface are the nucleic-acids. Source data are provided with this paper.

The DNA-binding protein 5h3r_A consists of 141 amino acids and 20 DNA-binding residues, as depicted in Fig. 5. SOFB accurately predicted all 20 binding residues of this protein. However, iDRNA-ITF predicted more false positives than we did, so our precision (Pre) was 0.188 higher than theirs. SOFB achieved the F1 of 0.909 and the Mathews correlation coefficient (MCC) of 0.898. In contrast, iDRNA-ITF resulted in F1 and MCC values of 0.784 and 0.766. This example demonstrates SOFB’s superior ability to detect true nucleic-acid-binding sites in sequences that prove challenging for alternative methods. Although SOFB predicts false positives for amino acids at various positions, they are predominantly spatially proximate to the nucleic acid. This finding suggests that SOFB can glean spatial structure information from one-dimensional sequence data, such as residue positions in three-dimensional space post-protein folding, and utilize it for nucleic-acid-binding residue identification.

The RNA-binding protein 6htu_A comprises 76 amino acids and 16 RNA-binding residues, as shown in Fig. 5. SOFB missed three binding sites on this protein chain, while iDRNA-ITF only missed predicting one binding site. However, its success in predicting more binding sites was at the cost of eighteen false-positive amino acids (the number of false positives for SOFB was one). This resulted in its F1 and MCC being 0.254 and 0.313 less than SOFB. The disparity between the two methods indicates that SOFB can reduce the number of predicted false positives when the number of positive examples identified is similar, which accounts for its superior performance.

### Interpretability analysis

**Fig. 6:**
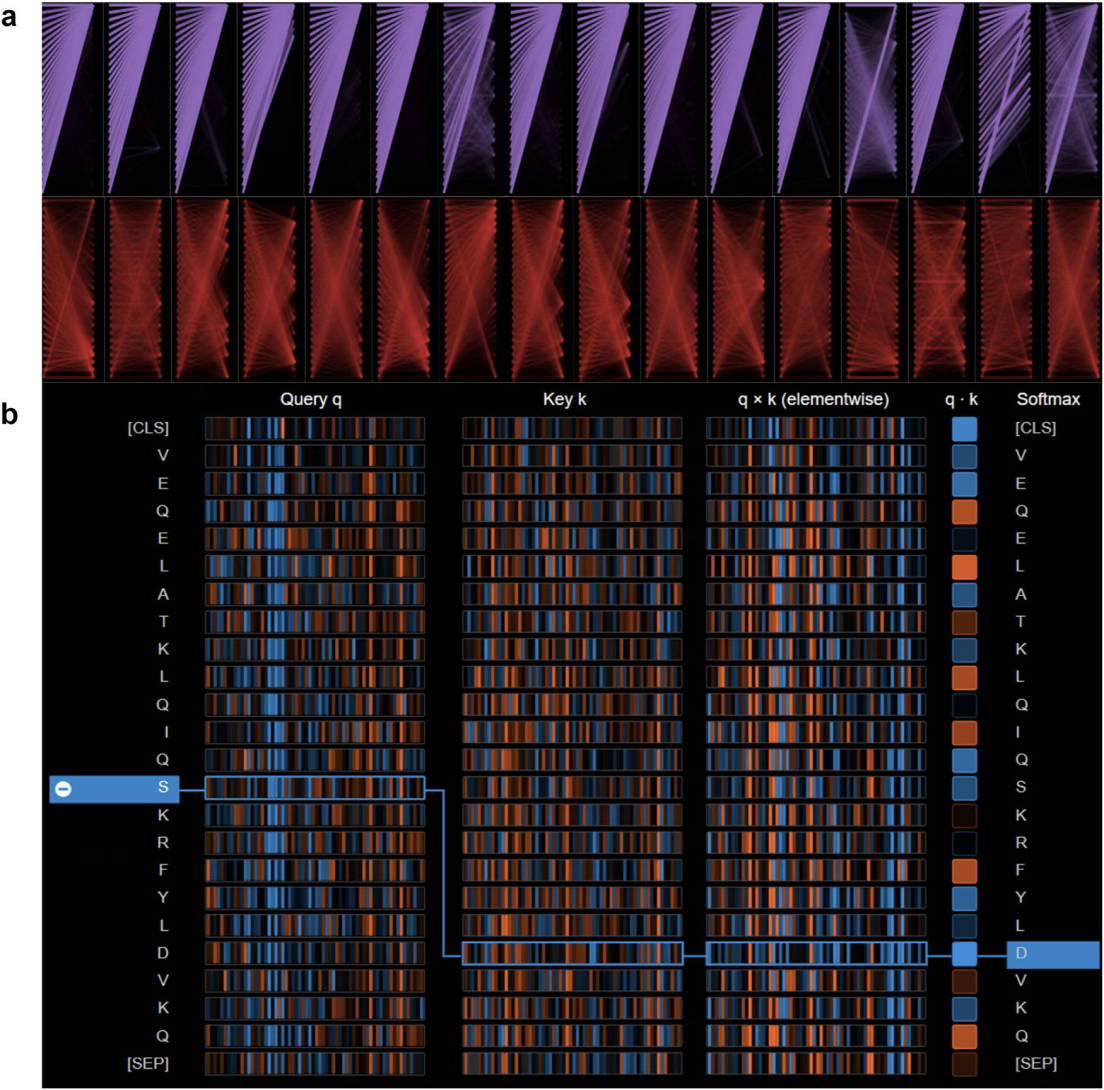
The general view of the different attentional distributions within the NABert model. **a** The attention heads distributed in two different layers, with the columns representing the attention heads in each layer, and the rows representing the particular heads in each layer. **b** The computation of the attention scores, where the first and second columns represent the query vector and the key vector, respectively. The vectors in the box show the two most-relevant tokens in the sequence. Source data are provided with this paper.

To investigate the extraction of dynamic semantic information from the NABert model, as illustrated in Fig. 6, we examined the attention weight implementation within the model. Figure 6a displays the two attention layers in the NABert model for the DNA-binding residue recognition task (Supplementary Fig. 9, Supplementary Note 10 for RNA task), encompassing the attention distribution of the fifth layer (in purple) and the fourteenth layer (in red). We observed that as the sequence iterates through the model’s layers, the attention scores of the attention heads within those layers transition from initially aggregating special characters to focusing on key regions of the sequence. These key regions contain residues that exert a substantial influence on the final nucleic-acid-binding residue recognition decision.

In addition, Fig. 6b illustrates the manner in which attention heads across various layers of the NABert compute attention scores for protein sequences within the DNA-binding test set (Supplementary Fig. 9, Supplementary Note 10 for RNA task). In this context, the Query vector (q) and Key vector (k) represent the respective Query and Key components within the model. Utilizing the Query and Key vectors, the attention scores between distinct markers can be determined through the attention formula. The figure denotes positive values in blue and negative values in orange. As an exemplar, we have selected the attention score for the sequence token ’S’, as computed by one of the attention heads in the 14th layer of the NABert. It is evident that the attention values between token ’S’ and other tokens in the chosen attention head do not exhibit substantial decay as the distance increases. This observation signifies that the NABert effectively maintains both long-distance and short-distance dependencies within the sequence, thereby ensuring its capacity to acquire profound semantic information pertaining to the sequence.

**Fig. 7:**
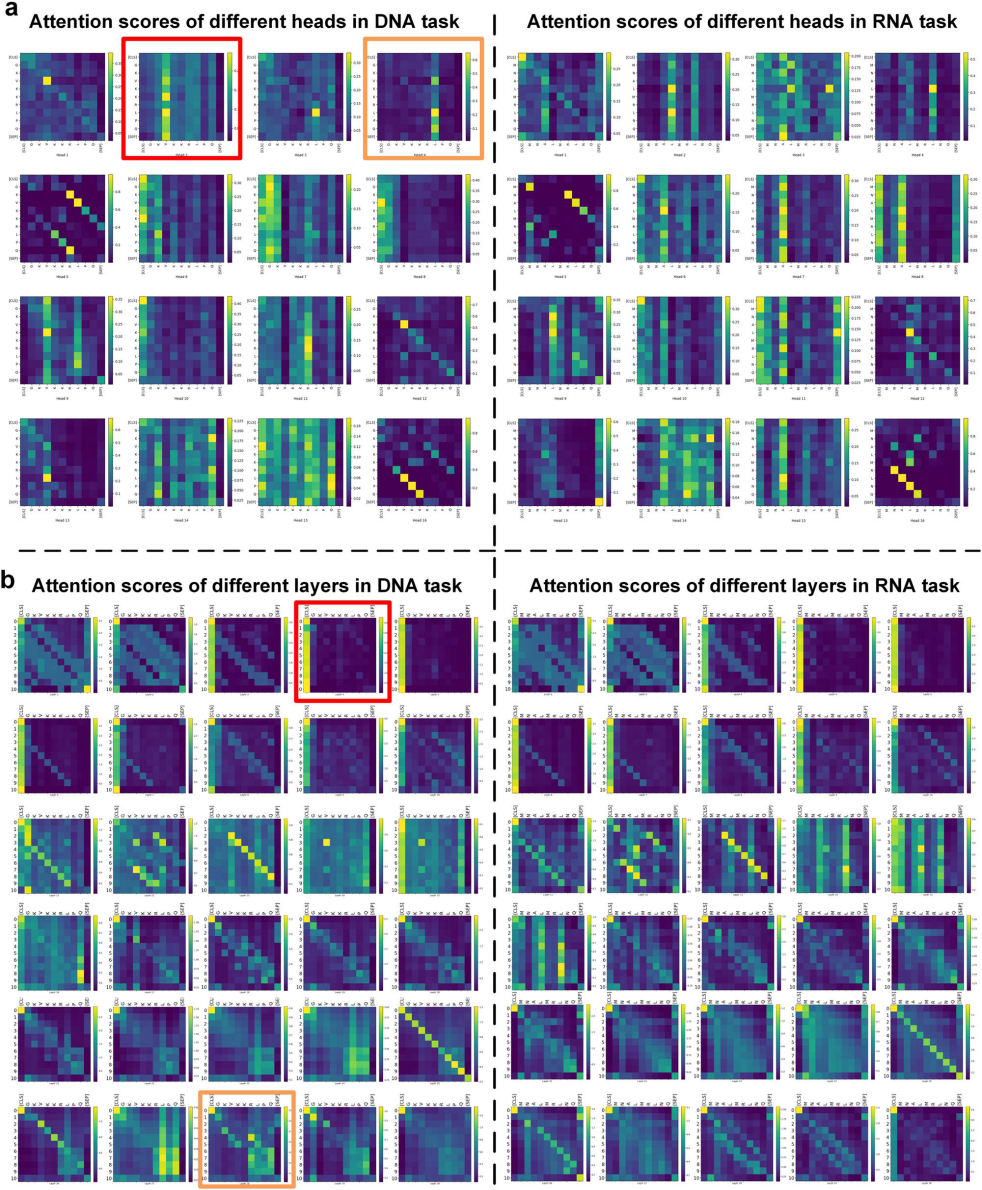
The visualization of the attention scores in the NABert model. **a** The attention maps of the 14th layer for DNA and RNA tasks, the graph in red box is the attention score of the second head, and the graph in orange box is the attention score of the fourth head. **b** The attention maps of all layers for DNA and RNA tasks, the graphs in red and orange boxes show the attention scores for the fourth and twenty-eighth layers, respectively. Source data are provided with this paper.

In the subsequent step, we generated attention maps utilizing the scores from all attention heads within each layer of the model. As depicted in Fig. 7a, heat maps representing attention scores for the second (in red) and fourth (in orange) attention heads in layer 14 are displayed. It can be observed that the majority of tokens in the second head target token ’V’, while most tokens in the fourth head target token ’L’. Distinct attention heads are clustered into separate regions, thereby augmenting the model’s ultimate predictive capability. Furthermore, we integrated the attention scores from all heads in each layer to produce attention maps for the thirty layers within the model. As illustrated in Fig. 7b, the fourth layer (in red) exhibits similarity to the vertical pattern^36^, suggesting attention towards individual tokens, typically ’SEP’ (special tokens indicating sentence endings) or ’CLS’ (full sequence used as input classifier representation of the special model token) tokens. Conversely, the twenty-eighth layer (in orange) resembles the block pattern^36^, denoting a consistent concentration on all tokens within the sequence. This outcome also demonstrates that, in most instances, not all attention results from the NABert model are necessarily meaningful. Moreover, we conducted statistical analysis on the attention maps. The experimental results are demonstrated in the Supplementary Fig. 10 of the Supplementary Note 10, suggesting that SOFB has the ability to concentrate more attention on biologically relevant positions, indicating its potential for discovering functional sites. These statistical results and hypothesis tests provide evidence for the effectiveness and potential interpretability of SOFB, offering different insights into the identification of functional sites.

We further conducted analysis of the features within SOFB. The SHAP analysis of the three features within SOFB for the DNA-binding residue prediction task and the RNA-binding residue task are illustrated in the Supplementary Fig. 11 and Supplementary Fig. 12 of the Supplementary Note 11. In summary, the SHAP visualization provides valuable insight into the contribution of various features to the prediction results, demonstrating that most feature values positively affect a specific number of samples.

### Application of predicted nucleic acid binding residues in molecular docking

**Fig. 8:**
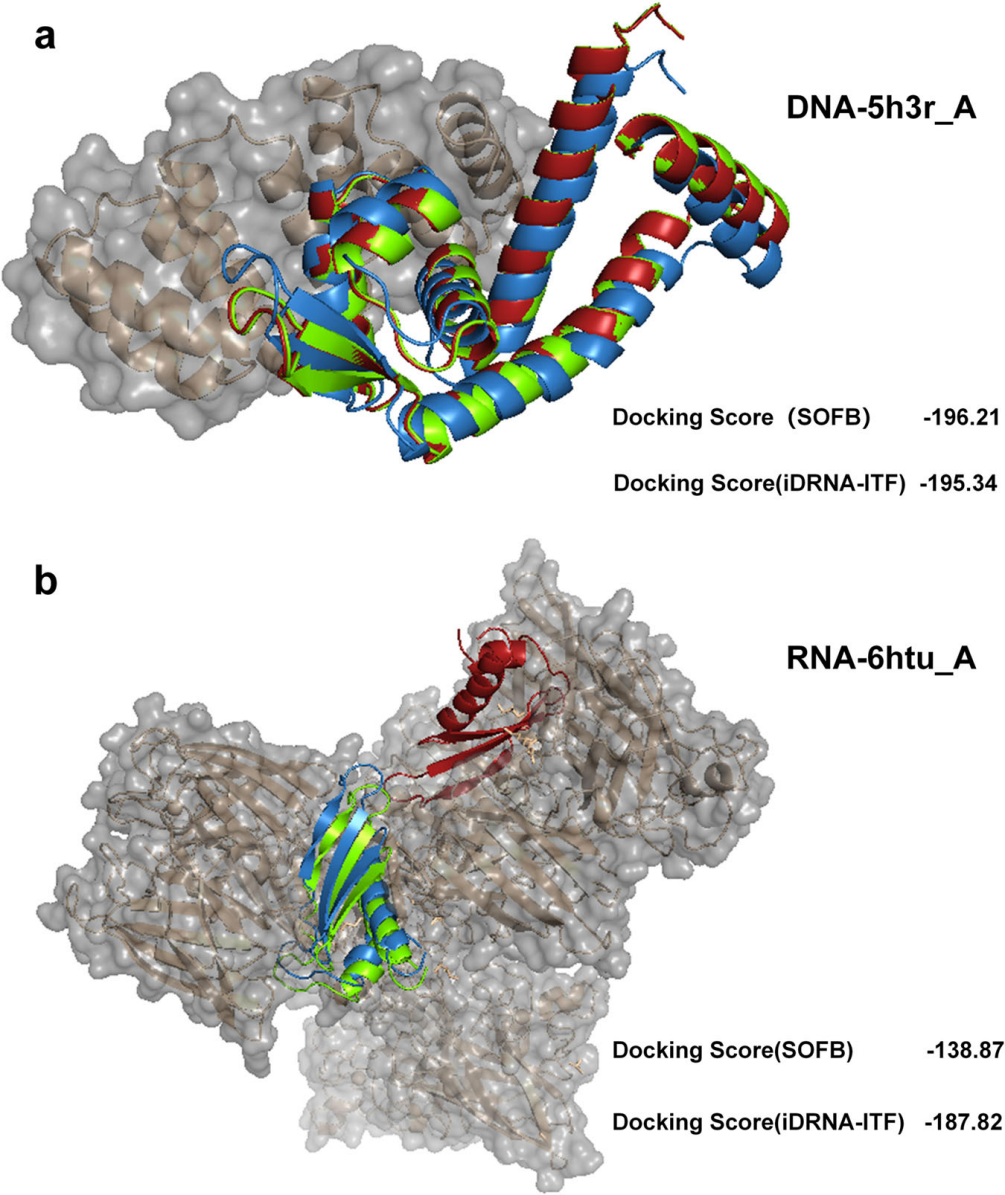
SOFB can perform molecular docking more accurately than the other methods. **a** The docking results of SOFB, iDRNA-ITF, and real labeling predictions in protein 5h3r_A, where the lower right corner is the Docking Score of the two methods. **b** The docking results of SOFB, iDRNA-ITF, and true label prediction in protein 6htu_A, where the bottom right corner is the Docking Score of the two methods. Green color in the figure represents the result of real label prediction, blue color indicates the result of SOFB prediction, and red color indicates the result of iDRNA-ITF prediction. Source data are provided with this paper.

Determining the docking location between proteins and nucleic acids is of great importance. To verify the feasibility of SOFB, we conducted experiment of docking position identification. In particular, we picked the two proteins 5h3r_A and 6htu_A with the highest MCC in the DNA and RNA binding test set, respectively, and used our SOFB along with iDRNA-ITF to predict the binding residues for comparison. We then employed HDOCK^37^, a protein-protein and protein-DNA/RNA docking server, for visualizing docking positions using protein sequences, binding nucleic acids structure obtained from PDB database^12^ and predicted binding residues as input. Finally, by calculating the docking scores through HDOCK, we can evaluate whether the model is capable of identifying docking positions by predicting the binding residues. The experimental results are summarized in Fig. 8.

Figure 8a illustrated the predicted docking position of SOFB and iDRNA-ITF for protein 5h3r_A that binds to DNA, where the green color in the figure represents the docking position predicted by the true label, blue color indicates the result of SOFB prediction, and red color indicates the result of iDRNA-ITF prediction. From the figure we can observe that although both SOFB and iDRNA-ITF can provide accurate residue predictions, our SOFB exhibits a better docking score with -196.21 (a more negative docking score means a more possible binding model). For Protein 6htu_A, despite iDRNA-ITF achieving a better docking score than our SOFB, SOFB outperforms iDRNA-ITF in predicting the true docking positions. As depicted in Fig. 8b, we can observe that the predictions of SOFB were highly consistent with the result predicted by true labels, while iDRNA-ITF exhibits a considerable distance from the true labels. It is also important to note that the docking score obtained from HDOCK should not be treated as the true binding affinity of two molecules because it has not been calibrated to the experimental data^37^. Therefore, these results and analyses will contribute to utilizing SOFB for docking position identifications, providing different insights into how proteins interact with nucleic acids. Moreover, we conducted additional experiments and employed RoseTTAFold^38^, the prototype of RoseTTAFoldNA^39^, to generate the extensive information of protein structures and integrate it as part of the bio-information into our SOFB to predict the nucleic acid-binding residues, as illustrated in the Supplementary Table 6 of the Supplementary Note 12. These results shows the incorporation of the RoseTTAFold method enriches the biological features of SOFB and enhances its performance.

## Discussion

In this study, we present SOFB, an ensemble deep learning model-based approach for the identification of nucleic-acid-binding residues in proteins. To better represent proteins if only sequence information is available, we explore several protein representation schemes from different perspectives, including learning dynamic biological contexts in the language learning model and learning general representations from multiple biological features. Then, we adopted the ensemble deep learning-based sequence net consisting of different convolutional layers and Bi-LSTM to characterize the various features. Finally, DNA-binding residues and RNA-binding residues in nucleic-acid-binding proteins are predicted by the fully-connected layer. In addition, we used ensemble learning to train multiple models to solve the sample imbalance problem. We performed training using datasets containing 573 DNA-binding proteins and 495 RNA-binding proteins and evaluated the models independently on two benchmark test sets. Our results show that SOFB can identify binding sites accurately and efficiently, over both independent test sets and multiple different datasets, and outperforms other state-of-the-art models such as iDRNA-ITF. Specifically, SOFB is able to accurately perform molecular docking, providing different insights into how proteins interact with nucleic acids. The case study also demonstrates that SOFB can learn part of the protein structure information from protein sequence information, which can potentially help in protein structure prediction. The interpretability analysis based on the attention scores demonstrates that SOFB can also explain some biological mechanisms, providing a basis for subsequent biomedical and drug discovery. In conclusion, SOFB is an excellent competitive model for nucleic acid binding residue recognition, providing a reliable tool for the study of proteins lacking structural information.

## Methods

### Problem formulation

For each protein sequence, we compute different features for each amino acid and get the probability that each amino acid within each protein sequence is a nucleic acid binding residue through the model. Thus, protein-nucleic-acid-binding site prediction is solved as a residue-level prediction. And the binding residue prediction task is applied as a binary classification problem, where the protein sequence is considered as the input and the output of the model is a L*2 matrix, and L denotes the length of the protein sequence and 2-dimension indicate whether the amino acid at each position is a binding residue or not.

### Protein representation schemes

#### Dynamic residue language embedding

In the protein domain, traditional protein representation methods often fail to capture the deep semantic meaning of the internal amino acid sequence, which is a major reason for their low efficiency in different tasks. However, recent language models have shown promising results in the protein field by using unsupervised learning to treat amino acids as words and capture the complex inter-sequence information. Therefore, we use two language model to extract as much semantic information within sequences as possible, where the sequences contain a total of 20 amino acids such as R, T, P, K, etc. The sequence ’ARRI’ is first segmented into individual tokens ’A’, ’R’, ’R’, ’I’, and then special tokens ’SEP’ and ’CLS’ are added to represent the sequence’s beginning and end, respectively. The vocabulary of the model contained 22 tokens^40^.

The language model comprises a bidirectional Transformer encoder model^41^ that performs both masked language modeling (MLM) and next sentence prediction (NSP) tasks. The core of the Transformer is the multi-head mechanism, which can capture unique features through Q, K, V vectors by using 16 different attention heads in 30 layers. In this way, we can obtain a richer representation by combining the features obtained by different heads:

1 $$Attention(Q,K,V)=softmax\left(\frac{Q{k}^{T}}{\sqrt{{d}_{k}}}\right)V$$

2 $$hea{d}_{i}=(Q{W}_{i}^{Q},K{W}_{i}^{K},V{W}_{i}^{V})$$

3 $$MultiHead(Q,K,V)=[hea{d}_{1}\oplus hea{d}_{2}\oplus \cdots \oplus hea{d}_{n}]{W}^{M},$$

where the four various Ws are weighting matrices, and *d*_*k*_ is the dimension of K, ⊕ represents the concatenation.

For a sequence: *a*_1_, *a*_2_, …, *a*_*n*_, the pre-training of the model will maximize the likelihood function *p*_*i*_ that predicts the *i*_*th*_ amino acids based on the amino acids before and after it^42^:

4 $${p}_{i}=p({a}_{i}| {a}_{1},\cdots \,,{a}_{i-1})+({a}_{i}| {a}_{i+1},\cdots \,,{a}_{n}).$$

In our task, considering the diversity of protein sequences, we used the ProtT5 model^18^, which has achieved effective performance in other tasks, to learn the representation of amino acids. The weight parameters of it were trained on the BFD and then fine-tuned on UniRef50^43^. As a result, the model can learn more general semantic properties of protein sequences due to the fact that learning has been performed on a larger number of protein sequences, in other words, when learning representations on unknown protein sequences, the model obtains representations that are more generalized compared to other methods. Finally, we take the output of the ProtT5 encoder as the features of the protein. Each amino acid can be expressed as a 1024-dimensional vector.

In addition, while the ProtT5 model improves the capability of the method partly, too many different tasks and too many protein sequences can further limit its ability. To overcome this limitation, we keep the number of layers as well as the number of heads of the original Bert language model and add a fully connected prediction layer. Then we fine-tune it with an epoch on the training sets to improve its ability on the nucleic-acid-binding residue recognition task, which is named NABert. During the fine-tuning process, the accuracy of the prediction results are used as an evaluation of the model. Moreover, we only need to extract the hidden states from the last layer of the model and drop the vector representation obtained from the special tokens ’CLS’ and ’SEP’ added before and after the sequence to generate the (n, 1024) matrix, where n is the length of the sequence markers and 1024 is the dimension of the vectors generated by NABert for each amino acid.

#### Evolutionary information

We use Position specific scoring matrix (PSSM)^44^ and hidden Markov models (HMMs)^45^ to obtain the evolutionary information of the sequences. For the PSSM, sequences are first scored by sequence matching in the NCBI’s non-redundant database^46^ with three iterations and E-value < 10^3^, which represents the possibility of amino acid interconversion. The scoring of each value x is then normalized to [0, 1] using sigmoid:

5 $$\bar{x}=\frac{1}{1+{e}^{-x}}.$$

The HMM is calculated by aligning the sequences in the uniclust30 database^47^. Its generated matrix consists of observed frequencies of 20 amino acids in homologous sequences, transition frequencies and local diversity. For each amino acid score, *h*_1_, ⋯  , *h*_20_ are the observed frequencies, *h*_21_, ⋯  , *h*_27_ are the transition frequencies, *h*_28_, *h*_29_, *h*_30_ represent the local diversity. We converted each value *h*_*i*_ to [0, 1] by the equation^2^ below:

6 $$\bar{{h}_{i}}=\frac{{h}_{i}}{10000}.$$

#### Physicochemical and biological characteristics

After that, to make our description of amino acids more specific, we choose three features of physicochemical characteristics: the number of atoms per amino acid (*pc*_1_), the electrostatic charge (*pc*_2_) and potential hydrogen bonding (*pc*_3_)^48^. The features of each type of amino acid are summarized in an array *phychem*_*type*_, and finally each feature will be normalized.

7 $$phyche{m}_{type}=\frac{[p{c}_{1},p{c}_{2},p{c}_{3}]-phyche{m}_{min}}{phyche{m}_{max}-phyche{m}_{min}}.$$

Then, to make our method include more biological properties of proteins and thus be more consistent with the biological characteristics, we also selected Relative amino acid propensity (RAA)^49^ and PKx^50^ to represent them. Specifically, RAA means the amino acid propensity for binding, which shows the relative difference in abundance between binding residues and the corresponding non-binding residues located on the protein surface. Positive (negative) values mean enrichment (depletion) among binding residues compared with the non-binding residues. Each amino acid has a fixed value. The PKx value for each amino acid type means the negative of the logarithm of the dissociation constant for any other group in the molecule.

Finally, to mark different amino acid species, we use one-hot encoding to distinguish between distinct amino acids. Our datasets have 20 types of amino acids, then we construct a 20-dimensional vector whose values are all 0, and only one position is assigned a value of 1 to represent one type of amino acid.

To summarize the above, we use the ProtT5 embeddings as well as the NABert embeddings as two inputs with feature matrices of (n, 1024). Additionally, we also combine PSSM, HMM, physicochemical biological characteristics, RAA, PKx, and one-hot as 75-dimensional features (n, 75). Thus, we construct the input features for three different aspects of SOFB.

### Ensemble deep learning model-based sequence network

#### Stacked convolutional module

Although the obtained protein representation contains rich global semantic information about the protein, it also contains much internal redundant information. We therefore built a stacked convolution module that is adapted on the image field’s ResNet^51^, to analyze the NABert embeddings to enable extraction of more complicated feature patterns and to guarantee the retention of accurate information. The stacked convolution module contains two types of blocks totaling 32 convolutional layers and a single convolutional layer at the beginning. There are 13 normal blocks and 3 extra connected blocks. Specifically, the normal blocks have two convolutional layers and the transmission system is directly summed, the extra connected blocks also have two convolutional layers, and the transmission system is passing the original input through a extra convolutional layer and then sums:

8 $${f}_{laye{r}_{i}}=({H}_{i} \, {f}_{laye{r}_{i-1}}+{b}_{i})+{f}_{laye{r}_{i-1}}$$

9 $${f}_{laye{r}_{i}}=({H}_{i} \, {f}_{laye{r}_{i-1}}+{b}_{i})+({H}_{e} \, {f}_{laye{r}_{i-1}}+{b}_{e}).$$

where ${f}_{laye{r}_{i}}$ and ${f}_{laye{r}_{i-1}}$ are the output and input of the *ith* convolutional layer, respectively. *H*_*i*_ and *b*_*i*_ represent the weight matrices and biases, and *H*_*e*_ and *b*_*e*_ represent the extra weight matrices and biases. The formula below represents the the calculation system in the extra connected blocks.

Through the handling of this module, every amino acid can get representation in a lower dimension, which contains better refined information than before. Besides, the information contained in the representation is constantly changing from ${f}_{a{s}_{1}}$ to ${f}_{a{s}_{n}}$ as it passes through the extra connected blocks within the network. Thus, we document the change via stacking them:

10 $${f}^{state}=\left[\left({f}_{a{s}_{1}}\right)\oplus \left({f}_{a{s}_{2}}\right)\oplus \cdots \oplus \left({f}_{a{s}_{n}}\right)\right]$$

11 $${f}_{R}=[ \, {f}_{laye{r}_{i}}\quad ,\quad {f}^{state}].$$

where *f* ^*state*^ is the description of the state change records of the NABert embedding, ⊕ is the concatenation operation, *f*_*R*_ is the NABert embeddings refined by this module. We believe that during the training process, the change of features also records specific information, so that the state will also serve as a support for the subsequent prediction.

#### Convolutional layers with different convolutional kernel sizes

To extract effective information at different scales of protein sequences described by biological features and NABert embeddings (*f*_*D*_), we set up four side-by-side convolutional layers^52^ with different convolutional kernel sizes to deal with them. For a sequence with feature dimension ’m’ and containing ’n’ amino acids, if the length of the convolution kernel is k, then the size of the convolution kernel *W*_*i*_ is k*m and the output dimension becomes a one-dimensional vector of length n-k+1. The outputs of multiple filters are concatenated to obtain the characteristics of each amino acid:

12 $${f}_{D}=[ \, {f}_{laye{r}_{i}},{f}_{Bio}]$$

13 $${f}_{GL}=p({W}_{1}{f}_{D})\oplus p({W}_{2}{f}_{D})\oplus \cdots \oplus p({W}_{q} \, {f}_{D}).$$

where *f*_*Bio*_ is the 75-dimension feature and *f*_*D*_ represents the features before convolution, *W*_*i*_ represents the weight matrix, *f*_*GL*_ represents the amino acid features after this module, ⊕ represents the concatenate operation, q represents the number of filters and p represents the pool operation.

This way, each feature representation includes both local features that describe amino acids in relation to their immediate neighbors and global features that describe the protein sequence.

#### Integrated multi-feature prediction module

To minimize the long-distance dependency problem caused by long protein sequences, we set up the integrated multi-feature prediction module based on LSTM^53^ with its gate mechanism (forget gate (f), input gate (i) and output gate (o)):

14 $${f}_{t}=\sigma ({W}_{f}\cdot [{h}_{t-1},{x}_{t}]+{b}_{f})$$

15 $${i}_{t}=\sigma ({W}_{i}\cdot [{h}_{t-1},{x}_{t}]+{b}_{i})$$

16 $${\widetilde{s}}_{t}=tanh({W}_{s}\cdot [{h}_{t-1},{x}_{t}]+{b}_{s})$$

17 $${s}_{t}={f}_{t}\circ {s}_{t-1}+{i}_{t}\circ {\widetilde{s}}_{t}$$

where *s*_*t*−1_ contains the state of all cells prior to the current one. $\widetilde{s}$ is the current cell state while *s*_*t*_ denotes the information that is passed to the next cell. In addition, *σ* is the logistic sigmoid function:

18 $${o}_{t}=\sigma ({W}_{o}\cdot [{h}_{t-1},{x}_{t}]+{b}_{o})$$

19 $${h}_{t}={o}_{t}\cdot tanh({s}_{t}).$$

The output of the current cell (*h*_*t*_) is obtained by combining the output gate (*o*_*t*_) with the information passed to the next cell (*s*_*t*_):

20 $$Integ=[ \, {f}_{ \!\!t},{i}_{t},{s}_{t},{o}_{t}].$$

The module includes three 64-unit LSTMs and a fully connected prediction layer. Two work with the state change records preserved by the NABert embeddings to mine the change patterns of features, and the other deals with the fusion of both global and local representations (*f*_*GL*_) and ProtT5 embeddings (*f*_*P*_). The fully connected layer receives the feature representation for categorization:

21 $$V=softmax\left[Integ\left( \, {f}_{ \!\!GL}\oplus {f}_{P}\right)\oplus Integ\left( \, {f}^{state}\right)\right]$$

where *f*_*P*_ is the ProtT5 embeddings and V is the prediction result.

#### Ensemble settings

To further improve the performance and stability of SOFB and mitigate the effects of positive and negative sample imbalance, we add parallel model ensemble settings. At present, Bagging is one of the mainstream ensemble methods that can incorporate the results of multiple side-by-side models, and decide the final sample results using the formulated rules. In this way, it can reduce the variance and improve the generalization performance of the model:

22 $$Var(mX)=E\left[{(mX-E[mX])}^{2}\right]={m}^{2}Var(X)$$

where X represents a sample, Var(X) is the variance, and E(X) represents the mean of X. Considering that it is a put-back sampling, the condition of independence between samples is not available. Under this condition, the variance can be described as:

23 $$Var\left(\frac{1}{n}{\sum }_{i=1}^{n}{X}_{i}\right)=\frac{\sigma }{{n}^{2}}+\frac{n-1}{n}\alpha {\sigma }^{2}$$

Therefore, an increase in classifiers and a decrease in inter-model correlation both lead to a reduction in variance. Motivated by this, we utilize ensemble learning for the protein dataset D. We build up n sets of learning processes, in which we take all positive samples as *D*_*p*_ and separate 1/n subsets *D*_*i*_ of *D*_*n*_ from the negative samples, *D*_*n*_ each time we learn. This process continues until all of the negative samples are learnt. This allows us to eventually obtain several classifiers simultaneously. After that, we sum the values obtained by these models for each sample in the test set and compute them by softmax to obtain the final recognition results.

### Parameter settings

In this work, all parameters of the SOFB implementation are set as follows. In the stacked convolutional layers module, we add 13 normal blocks and 3 extra connected blocks after a 1D convolutional layer, where each block contains two 1D convolutional layers. Each convolutional layer contains 32 filters with a convolutional kernel size of 3. The stride in the 3 extra connected blocks is set to 3 while the others are set to 2. Among the convolutional layers with different kernel sizes, we set four parallel convolutional layers to manage the biological features and the previous features with kernel sizes of 1, 3, 5, and 7 and filters set to 64. In the integrated multi-feature prediction, we set up three LSTM layers, each of which contains 64 units. We also add dropout layers with rates of 0.4 and 0.5. Finally, a fully connected layer with 2 hidden units is used with a softmax activation function. In the ensemble setting, we set the number of negative sample subsets to 4. Our model is trained on TensorFlow 2.7.0 and Keras 2.7.0, and the parameters of the hidden layer in the model adopts the default initialization of Keras. We employ an early stop controlled by the validation loss to avoid overfitting. Our network is trained end-to-end by the Adam optimizer with a batch size of 64 and a learning rate of 0.0005. In addition, the model is trained on an NVIDIA GeForce RTX 3090 GPU.

### Evaluation metrics

Nucleic acid-protein binding site prediction is solved as a binary classification problem. Consequently, we follow our previous studies using Recall (rec), Precision (pre), F1-score (F1) and Matthew’s correlation coefficient (MCC) as metrics to evaluate the performance of our method^5^. Calculation formulas are as follows:

Precision measures the probability of being positive in all samples that are predicted to be positive:

24 $$Precision=\frac{TP}{TP+FP}.$$

Recall measures the probability of being predicted as positive in a sample that is positive:

25 $$Recall=\frac{TP}{TP+FN}.$$

F1 measures the Harmonized average of the precision and recall:

26 $$F1=\frac{2\times Precision\times Recall}{Precsion+Recall}.$$

MCC uses all four elements of the confusion matrix TP, TN, FP and FN:

27 $$MCC=\frac{TP\times TN-FN\times FP}{\sqrt{(TP+FN)+(TP+FP)+(TN+FN)+(TN+FP)}},$$

where TP, FN, TN and FP denote the numbers of true positives, false negatives, true negatives and false positives, respectively. Specially, when the positive-negative sample ratio is not well balanced, F1 and MCC are more objective measures compared to Rec and Pre. The area under the receiver operating characteristic (ROC) curve (AUC), which reflects the most comprehensive prediction performance^54^, serves as another important evaluation metric.

### Reporting summary

Further information on research design is available in the Nature Portfolio Reporting Summary linked to this article.

### Supplementary information

Supplementary information file

Description of Additional Supplementary Files

Supplementary Data

Reporting Summary

## Data Availability

All datasets in this study are available on https://figshare.com/articles/online_resource/SOFB_figshare_rar/25499452^55^ and https://github.com/Encryptional/SOFB^56^. And the feature used can be generated following the usage tutorial on https://github.com/Encryptional/SOFB. In addition, the numerical source data for the graphs in the main figures can be found in the Supplementary Data file.

## Code availability

SOFB is implement by Python. All supporting source codes can be downloaded from https://github.com/Encryptional/SOFB, and the NABert models used and the SOFB models trained in this study are available on https://figshare.com/articles/online_resource/SOFB_figshare_rar/25499452.
