# Supplementary material for: SOFB is a comprehensive ensemble deep learning approach for elucidating and characterizing protein-nucleic-acid-binding residues
Source: Commun Biol. 2024 Jun 3;7:679. doi: 10.1038/s42003-024-06332-0 (PMC11148103; doi:10.1038/s42003-024-06332-0)
Supplement: Supplementary file 1 — Supplementary information file [file 42003_2024_6332_MOESM1_ESM.pdf]

SOFB is a Comprehensive Ensemble Deep Learning approach for Elucidating and Characterizing Protein-Nucleic-Acid-Binding Residues

Bin Zhang[1], Zilong Hou[1], Yuning Yang[2], Ka-chun Wong[3], Haoran Zhu[1*] and Xiangtao Li[1*]

[1]School of Artificial Intelligence, Jilin University, Changchun, China.
[2]Donnelly Centre for Cellular and Biomolecular Research, University of Toronto, Toronto, Canada.
[3]Department of Computer science, City University of Hong Kong, Hong Kong, SAR.

*Corresponding author(s). E-mail(s): zhuhr20@mails.jlu.edu.cn; lixt314@jlu.edu.cn;

Supplementary Material

Contents

1 **Supplementary Note 1: The details of the datasets used.** **1**

2 **Supplementary Note 2: The details of comparison with other language model** **1**

3 **Supplementary Note 3: The details of the heat maps in the correlation analysis** **2**

4 **Supplementary Note 4: The experiment of chain interactions** **3**

5 **Supplementary Note 5: The details of comparison with baseline methods** **5**

6 **Supplementary Note 6: The result of SOFB on different protein family** **6**

7 **Supplementary Note 7: The results of Ablation Study** **9**

8 **Supplementary Note 8: The results of SOFB on other datasets** **10**

9 **Supplementary Note 9: The details of Case Study** **12**

10 **Supplementary Note 10: The details of Interpretability Analysis** **17**

11 **Supplementary Note 11: The details of SHAP analysis** **20**

12 **Supplementary Note 12: The results of SOFB that incorporate with the RoseTTAFold method** **23**

1 Supplementary Note 1: The details of the datasets used.

Supplementary Table 1: The details of the datasets used. From top to bottom, the name of the datasets, the number of protein sequences in the datasets, the number of nucleic-acid-binding residues in the datasets and the number of non-nucleic-acid-binding residues in the datasets. Source data are provided with this paper.

**DNA-protein**		**RNA-protein**	
**DNA-573_Train**	**DNA-129_Test**	**RNA-495_Train**	**RNA-117_Test**
**573**	**129**	**495**	**117**
**14479**	**2240**	**14609**	**2031**
**145404**	**35275**	**122290**	**35314**

2 Supplementary Note 2: The details of comparison with other language model

Supplementary Figure 1 illustrates the overall prediction performance of SOFB across different feature characterizations. The Supplementary Figure 1 indicates that despite fine-tuning, the performance of ESM (esm1_t34_670M_UR100) [1] shows negligible improvement. In terms of recognizing DNA and RNA binding residues, the AUCs improved marginally by 0.001 and 0.003, reaching 0.898 and 0.802, respectively. However, these enhancements remain insufficient compared to the bio-language learning model initially employed. Notably, the performance of the latest ESM2 (esm2_t12_35M_UR50D) [2] surpasses both ESM and fine-tuned ESM, exhibiting superior metrics across the board, particularly in F1 and MCC. Specifically, ESM2 achieved AUCs of 0.902 and 0.822 for the DNA and RNA tasks, respectively. However, after fine-tuning, ESM2's performance witnessed a decline,

with AUCs dropping to 0.869 and 0.780, representing decrements of 0.032 and 0.042, respectively. This decline might stem from the ESM2 model's reduced suitability for fine-tuning compared to ESM, possibly influenced by variations in certain layer parameters that led to a departure from its original performance level.

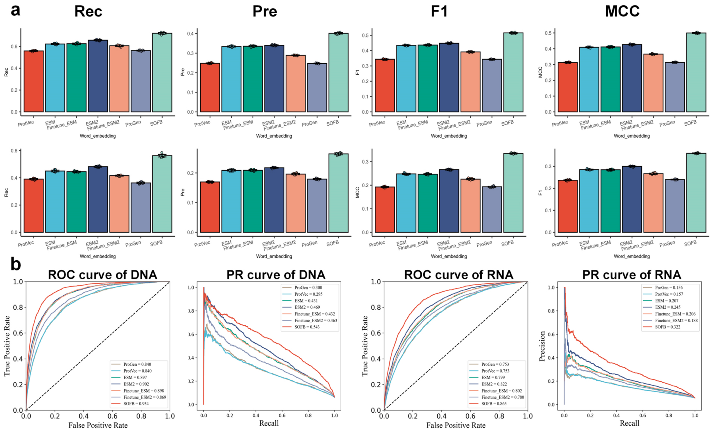

Supplementary Figure 1: (a) shows the average Recall (Rec), Precision (Pre), F1, MCC of ten runs of six dynamic contextual embeddings (ProtVec, ESM, Finetune_ESM, ESM2, Finetune_ESM2, ProGen) on DNA-binding test set and RNA-binding test set; (b) provides ROC curves with AUC values, PR curves with AP values for DNA-binding residue and RNA-binding residue predictions, respectively, where SOFB performs best by both metrics. Source data are provided with this paper.

3 Supplementary Note 3: The details of the heat maps in the correlation analysis

Supplementary Figure 2 exhibits the feature correlation of SOFB using different feature characterizations. Upon analysis of the correlation heatmaps, we observe that SOFB consistently demonstrates superior performance compared

to all other dynamic methods in the classification of amino acids and the segregation of amino acids within a sequence into two distinct groups, crucial for subsequent identification of nucleic acid binding residues. It's noteworthy that in the context of recognizing DNA binding residues, the heatmap derived from fine-tuned ESM2 lacks discriminatory patterns entirely, attributing to the stark decline in performance observed. Overall, the feature construction strategy of SOFB surpasses other methods, showcasing the effectiveness and robustness of SOFB.

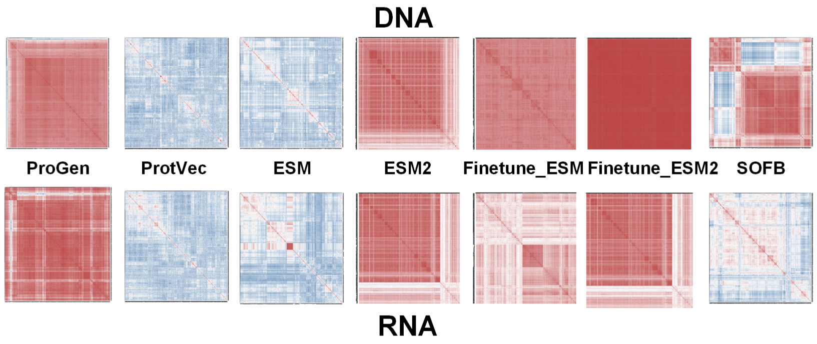

Supplementary Figure 2: Heat maps of correlation analysis of six dynamic contextual embeddings (ProtVec, ESM, Finetune_ESM, ESM2, Finetune_ESM2, ProGen) and SOFB on DNA-binding test set and RNA-binding test set are given. Source data are provided with this paper.

4 Supplementary Note 4: The experiment of chain interactions

We conducted another experiment to investigate the effect of chain interactions on the prediction performance of our SOFB. In particular, we adjusted the number of protein chains in the training sets and obtained different number of protein chains with five experimental groups of 100, 200, 300, 400, and

all protein chains. The experimental results are tabulated in Supplementary Table 2. From the experimental results, it can be observed that in the DNA binding residue recognition task, as the number of protein chains increases, the interactions between protein chains are enhanced, which leads to the increment of the results.

Moreover, in the RNA-binding residue identification task, the prediction performance also improves with the increasing number of protein chains. In addition, the ROC curves of the experimental results are illustrated in Supplementary Figure 3, from which we can observe that although the number of protein chains in the training set had a slight impact on the performance of SOFB, an increase in the number of chains enhanced the interaction between protein chains, thereby further improving the predictive capability of SOFB.

Supplementary Table 2: From left to right each column shows the number of protein chains, the number of amino acids, the number of binding residues, the number of non-binding residues, Rec, Pre, F1, MCC, AUC, respectively. Source data are provided with this paper.

			DNA					
**Pro_nums**	**Res_nums**	**Pos_nums**	**Neg_nums**	**Rec**	**Pre**	**F1**	**MCC**	**AUC**
**100**	**34564**	**3127**	**31437**	**0.671**	**0.388**	**0.492**	**0.470**	**0.920**
**200**	**88154**	**6327**	**81827**	**0.689**	**0.394**	**0.501**	**0.482**	**0.925**
**300**	**120841**	**9207**	**111634**	**0.700**	**0.404**	**0.512**	**0.493**	**0.931**
**400**	**142075**	**11591**	**130484**	**0.704**	**0.413**	**0.520**	**0.502**	**0.932**
**573**	**159883**	**14479**	**145404**	**0.730**	**0.404**	**0.520**	**0.505**	**0.934**
			**RNA**					
**100**	**32201**	**3808**	**28393**	**0.511**	**0.269**	**0.352**	**0.321**	**0.842**
**200**	**74548**	**7224**	**67324**	**0.503**	**0.264**	**0.346**	**0.314**	**0.848**
**300**	**111533**	**10273**	**101260**	**0.534**	**0.273**	**0.361**	**0.333**	**0.859**
**400**	**129106**	**12732**	**116374**	**0.549**	**0.270**	**0.362**	**0.335**	**0.860**
**495**	**136899**	**14609**	**122290**	**0.585**	**0.258**	**0.358**	**0.337**	**0.865**

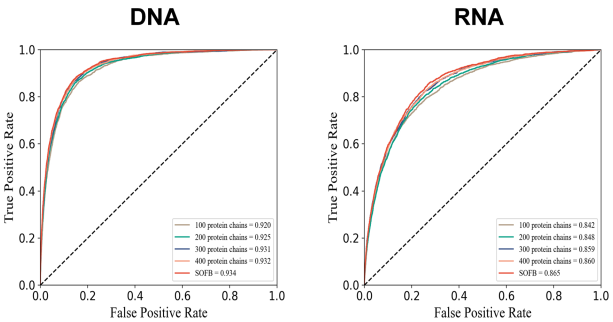

Supplementary Figure 3: provides the ROC curves for experiments controlling the number of protein chains (100, 200, 300, 400, all) in the training sets for both tasks. Source data are provided with this paper.

5 Supplementary Note 5: The details of comparison with baseline methods

To comprehensively demonstrate the effectiveness of our proposed SOFB, we combined multiple performance metrics and depicted them in a violin plot (Supplementary Figure 4), where each data point (represented by a triangle) signifies a predictive metric for the respective model. It is evident that our SOFB exhibits superior overall performance compared to other methods. The Supplementary Table 3 is given for a detailed comparison.

Supplementary Table 3: Showing the values of Pre, Rec, F1, MCC, AUROC for combining different baseline methods with SOFB, where the results of SOFB outperforms all other methods. Source data are provided with this paper.

				DNA					
**Methods**	**COACH-D**	**SVMnuc**	**NucBind**	**DNABind**	**TargetDNA**	**DNAPred**	**NCBRPred**	**GraphBind**	**iDRNA-ITF**	**SOFB**
**Rec**	**0.324**	**0.316**	**0.323**	**0.601**	**0.417**	**0.396**	**0.312**	**0.567**	**0.5**	**0.73**
**Pre**	**0.36**	**0.371**	**0.373**	**0.346**	**0.28**	**0.353**	**0.392**	**0.2**	**0.389**	**0.404**
**F1**	**0.341**	**0.341**	**0.346**	**0.44**	**0.335**	**0.373**	**0.348**	**0.295**	**0.438**	**0.52**
**MCC**	**0.302**	**0.304**	**0.309**	**0.411**	**0.291**	**0.332**	**0.313**	**0.292**	**0.401**	**0.505**
**AUC**	**0.761**	**0.812**	**0.797**	**0.858**	**0.825**	**0.845**	**0.823**	**0.855**	**0.883**	**0.934**

				RNA						
**Methods**	**COACH-D**	**SVMnuc**	**NucBind**	**aaRNA**	**NucleicNet**	**RNABindRPlus**	**NCBRPred**	**GraphBind**	**iDRNA-ITF**	**SOFB**
**Rec**	**0.221**	**0.231**	**0.231**	**0.484**	**0.371**	**0.273**	**0.135**	**0.439**	**0.349**	**0.585**
**Pre**	**0.252**	**0.24**	**0.235**	**0.166**	**0.201**	**0.227**	**0.3**	**0.111**	**0.235**	**0.258**
**F1**	**0.235**	**0.235**	**0.233**	**0.247**	**0.261**	**0.248**	**0.187**	**0.177**	**0.281**	**0.358**
**MCC**	**0.195**	**0.192**	**0.189**	**0.214**	**0.216**	**0.202**	**0.172**	**0.164**	**0.236**	**0.337**
**AUC**	**0.663**	**0.729**	**0.715**	**0.771**	**0.788**	**0.717**	**0.667**	**0.738**	**0.76**	**0.865**

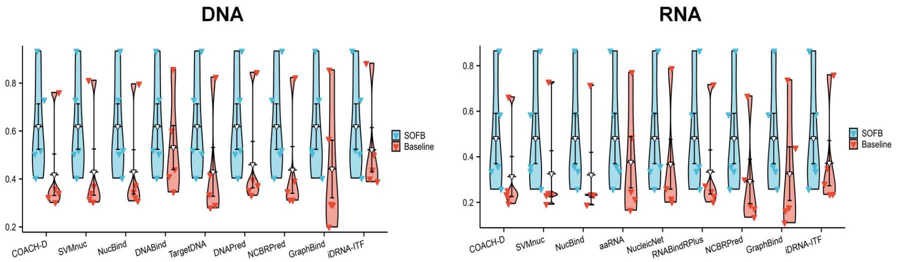

Supplementary Figure 4: provides a comparison of SOFB with other state-of-the-art algorithms on the DNA (RNA)-binding test sets, where other algorithm results are reported in [3]. It shows violin plots depicting multiple performance metrics of different baseline methods along with SOFB, where SOFB outperforms all other methods. The triangle markers measn Recall (Rec), Precision (Pre), F1 score, Matthews correlation coefficient (MCC) and Area Under the Curve (AUC) (n=5). Source data are provided with this paper.

6 Supplementary Note 6: The result of SOFB on different protein family

We conducted experiments on evaluating the performance of our SOFB on predicting nucleic acid binding residues of proteins across different protein families(protein family). We primarily utilized the protein binding residue dataset obtained from BioLip [4] in our study. Although we cannot directly obtain the data classified according to protein family from BioLip, we categorized the proteins in the BioLip test set based on the provided protein IDs by indexing in InterPro [5], thereby classifying the proteins according to their protein families. We then evaluated the performance of SOFB across different protein families using MCC as the evaluation metrics.

The experimental results were summarized in the top of the Supplementary Figure 5, where the top seven protein families that were best characterized were illustrated. From the Supplementary Figure 5, we can observe that SOFB performed best on the Bacterial regulatory proteins, tetR family (PF00440), which represents a DNA-binding domain with a helix-turn-helix (HTH) structure.

MerR HTH family regulatory protein (PF13411) has a winged helix-turn-helix (wHTH) structural domain. MarR family (PF01047), Myb-like DNA-binding domain (PF00249) also belongs to the clan of HTH. Therefore, we infer that SOFB has concentrations on DNA-binding residues with protein clan for HTH structure.

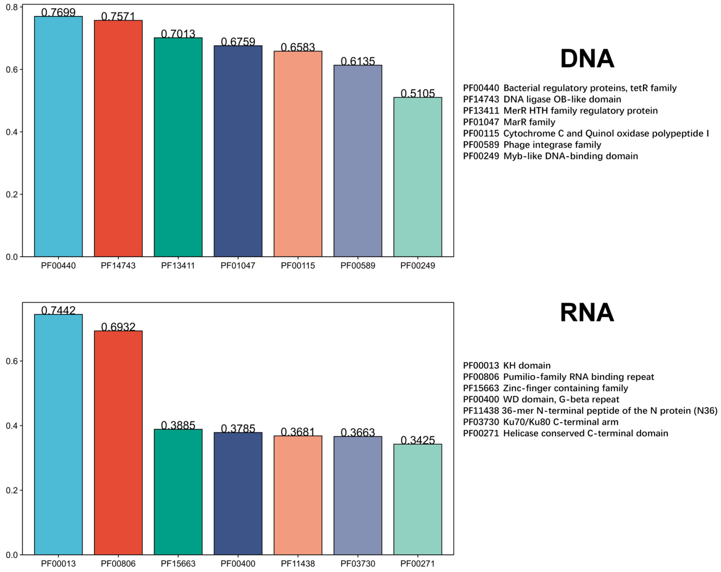

Supplementary Figure 5: SOFB predicts results for different protein family within the DNA and RNA-binding test datasets (using MCC as a metric), and shows the protein family among them with the best results, respectively. Source data are provided with this paper.

In terms of RNA-binding residues prediction task as illustrated in the bottom of the Supplementary Figure 5, SOFB performs best on the KH domain (PF00013), which presents in a wide variety of nucleic acid-binding proteins. Pumilio-family RNA binding repeat (PF00806), the Puf domains that usually occur as a tandem repeat of 8 domains, was also accurately inferred for its binding residues by SOFB. Unfortunately, SOFB exhibited limited performance in the RNA binding prediction task within other protein families. We

speculate that it is due to the presence of multiple proteins or more repeats with in protein families PF00013 and PF00806, resulting in that SOFB learns more informative features of such proteins. Overall, in both DNA binding and RNA binding prediction tasks, SOFB has demonstrated excellent performance in certain protein families.

7 Supplementary Note 7: The results of Ablation Study

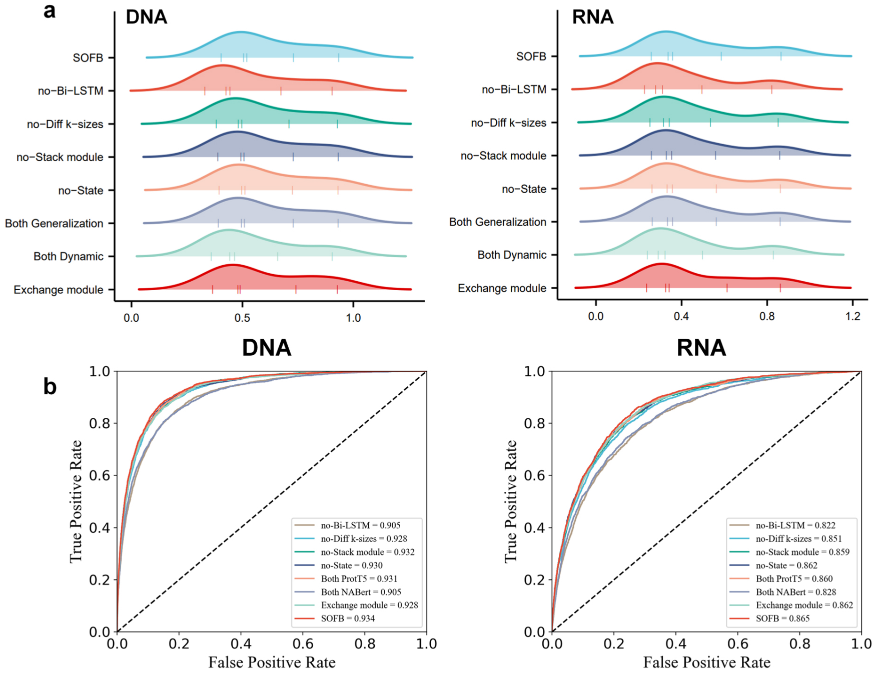

Supplementary Figure 6: (a) provides ablation experiments of SOFB testing on nucleic-acid-binding test sets in the different settings, from top to bottom, SOFB, setting (a), setting (b), setting (c) and setting (d), respectively. Then setting (e), setting (f) and setting (g), where the setting (a, b, c and d) and (e, f, g) are ablation of the structure and feature matching modules, respectively. (b) shows the ROC curves of the ablation experiment on DNA and RNA-binding residues prediction tasks, from top to bottom, a(no-Bi-LSTM), b(no Diff-k sizes), c(no Stack module) and d(no-State), respectively. Then e(Both ProtT5),f(Both NABert), e(Exchange module) and SOFB, where the setting (a, b, c and d) and (e, f, g) are ablation of the structure and feature matching modules, respectively. (n=5) Source data are provided with this paper.

8 Supplementary Note 8: The results of SOFB on other datasets

We have conducted additional experiment to explore and compare the predictive capabilities of SOFB. Firstly, we collected YFK16, YK17 and MW15 test datasets from [6], and each dataset consisted of 2 subsets for protein–DNA and protein–RNA binding. Subsequent evaluations of the nucleicacid-binding residues prediction performance on these tissue datasets with metric AUC involved SOFB, along with other baseline models including DRNApred, COACH-D, SVMnuc, Nucbind and iDRNA-ITF. As illustrated in Supplementary Table 4, we can observe that SOFB performs best comparing with the other methods on all datasets. For instance, in DNA binding prediction, none of the methods achieved a performance exceeding 0.9. Both DRNApred and COACH-D fell short of 0.8. In contrast, our SOFB exhibited the highest prediction AUC, reaching a remarkable 0.949. This underscores the effectiveness of SOFB in recognizing amino acid binding patterns. Furthermore, in RNA binding prediction, a decrease in performance was observed for all methods except SOFB. For example, DRNApred yielded a prediction AUC of only 0.467 on the MW15 dataset, while COACH-D achieved merely 0.579 on the same dataset. However, our SOFB maintained its superior predictive capability, attaining the highest AUC of 0.94. These findings demonstrate that SOFB possesses stronger effectiveness and robustness in predicting nucleic-acid binding in both DNA and RNA contexts.

Supplementary Table 4: The names of different datasets, the cutoff defined for different datasets, the AUROC predicted by DRNApred, COACH-D, SVM-nuc, Nucbind and iDRNA-ITF on YFK16(3.5 Å), YFK16(5 Å), YK17, MW15. Source data are provided with this paper.

	**DNA_binding_prediction**				**RNA_binding_prediction**			
**Datasets**	**YFK16**	**YFK16**	**YK17**	**MW15**	**YFK16**	**YFK16**	**YK17**	**MW15**
**Cutoff**	**3.5**	**5**	**3.5**	**5**	**3.5**	**5**	**3.5**	**5**
**DRNApred**	**0.757**	**0.738**	**0.77**	**0.725**	**0.687**	**0.666**	**0.67**	**0.467**
**COACH-D**	**0.771**	**0.764**	**0.69**	**0.713**	**0.711**	**0.732**	**0.64**	**0.579**
**SVMnuc**	**0.833**	**0.821**	**0.80**	**0.829**	**0.784**	**0.785**	**0.74**	**0.789**
**Nucbind**	**0.834**	**0.822**	**0.81**	**0.830**	**0.801**	**0.803**	**0.75**	**0.794**
**iDRNA-ITF**	**0.870**	**0.855**	**0.859**	**0.870**	**0.871**	**0.840**	**0.839**	**0.839**
**SOFB**	**0.949**	**0.919**	**0.947**	**0.945**	**0.940**	**0.909**	**0.921**	**0.917**

Besides, due to the limited size of the test datasets, we ultimately selected the YK17 training dataset as our large-scale benchmark dataset. In particular, we employed CD-HIT method to eliminate protein sequences with identity exceeding 30%, resulting in a final dataset of 464 proteins with totaling 106,081 that binds to DNA and 416 RNA binding proteins with totaling 95,020 amino acids. Subsequently, we compared the predictive performance of SOFB with iDRNA-ITF [3] on this dataset to validate the performance of SOFB on large-scale datasets.

Supplementary Table 5: The number of protein entries within the large dataset used, the number of binding residues versus non-binding residues, and the performance of SOFB and iDRNA-ITF on the dataset shows that the results of SOFB are superior to iDRNA-ITF, which is currently the best performer. Source data are provided with this paper.

	Methods	Protein_num	binding_num	non-binding_num	Pre	Rec	F1	MCC	AUC
DNA	iDRNA-ITF	464	7823	98258	0.625	0.442	0.518	0.480	0.897
Binding	SOFB	464	7823	98258	0.885	0.383	0.534	0.536	0.949
RNA	iDRNA-ITF	416	3202	91818	0.677	0.283	0.399	0.409	0.911
Binding	SOFB	416	3202	91818	0.860	0.148	0.253	0.313	0.913

The experimental results were summarised in Supplementary Table 5. In terms of DNA binding resisues prediction, From the table, we can observe that SOFB outperformed iDRNA-ITF by a margin on the large-scale dataset.

For instance, SOFB achieved an AUC improvement of over 5% and a precision improvement of over 10%. In the RNA binding prediction task, we observed a narrower gap between SOFB and iDRNA-ITF. Both models exhibited similar AUC values, but SOFB continued to outperform iDRNA-ITF in precision, reaching a remarkable precision score of 0.86. Overall, our SOFB model maintains its strong effectiveness and remains highly competitive even on larger-scale datasets.

9 Supplementary Note 9: The details of Case Study

We have conducted additional analyses and employ a more consistent criterion for proteins selection, where the three protein chains with the highest MCC score obtained by the best two models (SOFB and iDRNA-ITF) were selected. For the DNA task, the top three proteins are 5h3r_A, 6c31_A, 6enb_A, and for the RNA task, they are 6htu_A, 5www_A and 5wzg_A. We compared the predictive results of SOFB and iDRNA-ITF and visualized the nucleic-acid-binding residues of these proteins in the DNA and RNA-binding, respectively. The visualization of the protein chains are illustrated in Supplementary Figure 7 and Supplementary Figure 8.

The DNA-binding protein 5h3r_A consists of 141 amino acids and 20 DNA-binding residues, as depicted in Supplementary Figure 7. Both SOFB and iDRNA-ITF accurately predicted all 20 binding residues of the protein. However, iDRNA-ITF predicted more false positives than we did, so our precision (Pre) was 0.188 higher than theirs. SOFB achieved the F1 of 0.909 and the Mathews correlation coefficient (MCC) of 0.898. In contrast, iDRNA-ITF resulted in F1 and MCC values of 0.784 and 0.766.

The DNA-binding protein 6c31_A consists of 195 amino acids and 17 DNA-binding residues. SOFB located 15 binding residues with two residues lost. iDRNA- ITF found 16 binding residues with one residue lost. However, iDRNA-ITF predicted more false-positive amino acids (6 for iDRNA-ITF compared to 2 for SOFB). Finally, SOFB achieved a precision (Pre) of 0.882, recall (Rec) of 0.882, F1-score of 0.882, and Matthews correlation coefficient (MCC) of 0.871. In contrast, the Pre, Rec, F1 and MCC values of iDRNA-ITF were 0.727, 0.941, 0.821 and 0.809, respectively.

The DNA-binding protein 6enb_A consists of 184 amino acids and 17 DNA-binding residues. SOFB accurately predicted 15 binding residues of this protein, achieving a precision (Pre) of 0.833, recall (Rec) of 0.882, F1-score of 0.857, and Matthews correlation coefficient (MCC) of 0.843. In contrast, iDRNA-ITF identified only 13 out of the 10 DNA-binding residues, with Pre, Rec, F1, and MCC values of 0.813, 0.765, 0.788, and 0.767, respectively.

The three protein chains with the best results within SOFB demonstrates SOFB's superior ability to detect true nucleic-acid-binding sites in sequences that prove challenging for alternative methods. Although SOFB predicts false positives for amino acids at various positions, they are predominantly spatially proximate to the nucleic acid. This finding suggests that SOFB can glean spatial structure information from one-dimensional sequence data, such as residue positions in three-dimensional space post-protein folding, and utilize it for nucleic-acid-binding residue identification.

The RNA-binding protein 6htu_A comprises 76 amino acids and 16 RNA-binding residues, as shown in Supplementary Figure 8. SOFB missed three binding sites on this protein chain, while iDRNA-ITF only missed predicting one binding site. However, its success in predicting more binding sites was at

the cost of eighteen false-positive amino acids (the number of false positives for SOFB was one). This resulted in its F1 and MCC being 0.254 and 0.313 less than SOFB.

The RNA-binding protein 5www_A comprises 94 amino acids and 24 RNA-binding residues. The RNA-binding protein 5www_A consists of 94 amino acids and 24 RNA-binding residues. SOFB and iDRNA-ITF identified 19, 17 binding residues and 3, 9 false positives for this protein, respectively. The Pre of SOFB was 0.864, Rec was 0.792, F1 was 0.826, and MCC was 0.771. The Pre, Rec, F1, and MCC of iDRNA-ITF were 0.654, 0.708, 0.680, and 0.565, respectively.

The RNA-binding protein 5wzg_A comprises 534 amino acids and 44 RNA-binding residues, as shown in Supplementary Figure 8. SOFB identified 39 binding residues for this protein, with 22 false positives, resulting in a Pre of 0.639, Rec of 0.886, F1 of 0.743, and MCC of 0.727. In comparison, iDRNA-ITF detected 31 RNA-binding residues with 42 false positives, yielding Pre, Rec, F1, and MCC values of 0.425, 0.705, 0.430, and 0.495, respectively.

From these results, it is evident that SOFB exhibits more pronounced advantages in the recognition of RNA binding residues. Its superiority lies in its ability to mitigate false positives when identifying a comparable number of correct examples. This factor contributes to its enhanced performance in this particular task.

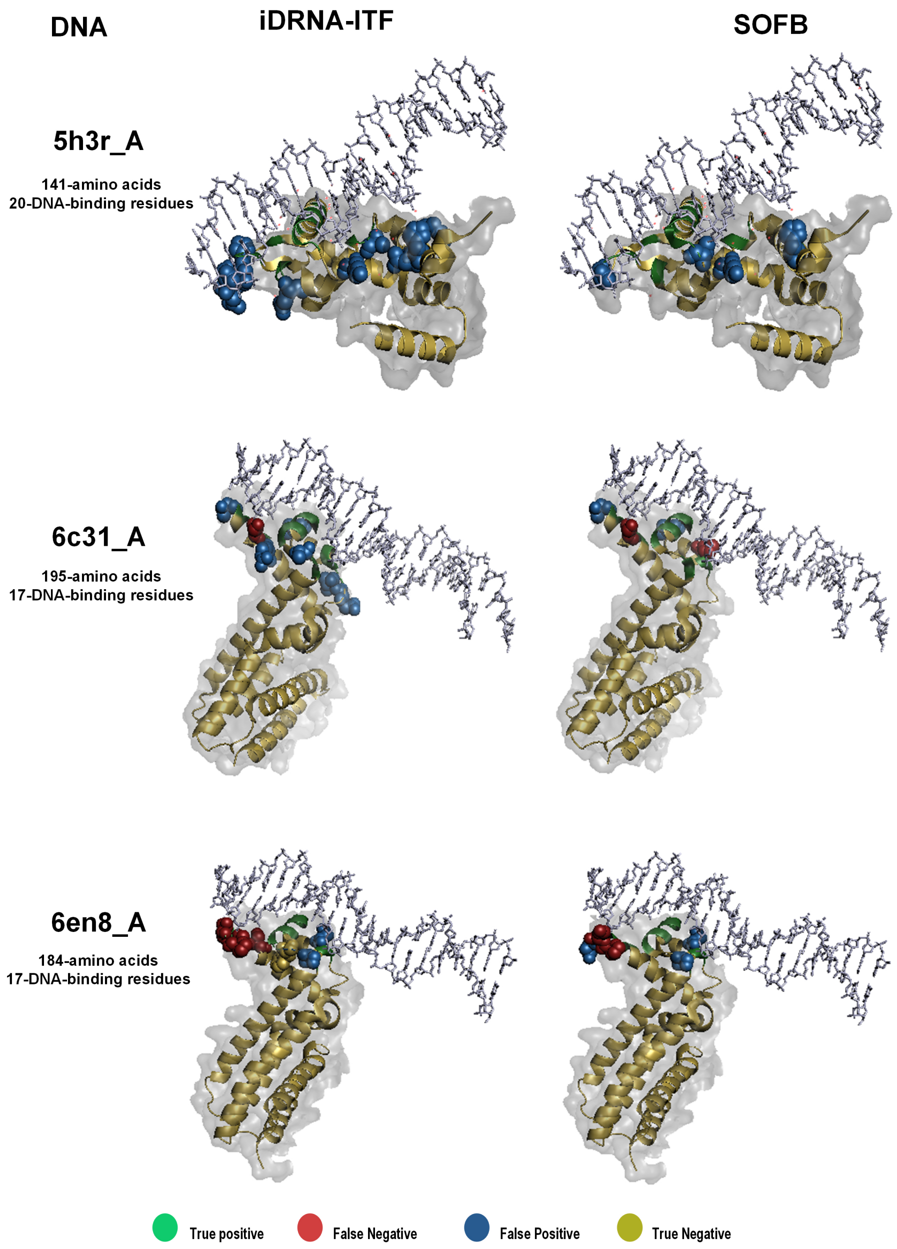

Supplementary Figure 7: Visualization of two cases predicted by SOFB and the suboptimal method iDRNA-ITF. For DNA-129_Test the top three MCC ranked proteins were selected: 5h3r_A, 6c31_A, 6enb_A, showing the results predicted by iDRNA-ITF (left) and SOFB (right). The red spheres are residues predicted to be false-negative, the blue spheres are residues predicted to be false-positive, green represents true-positive residues, and yellow represents true-negative residues. Outside the white protein surface are nucleic acids.

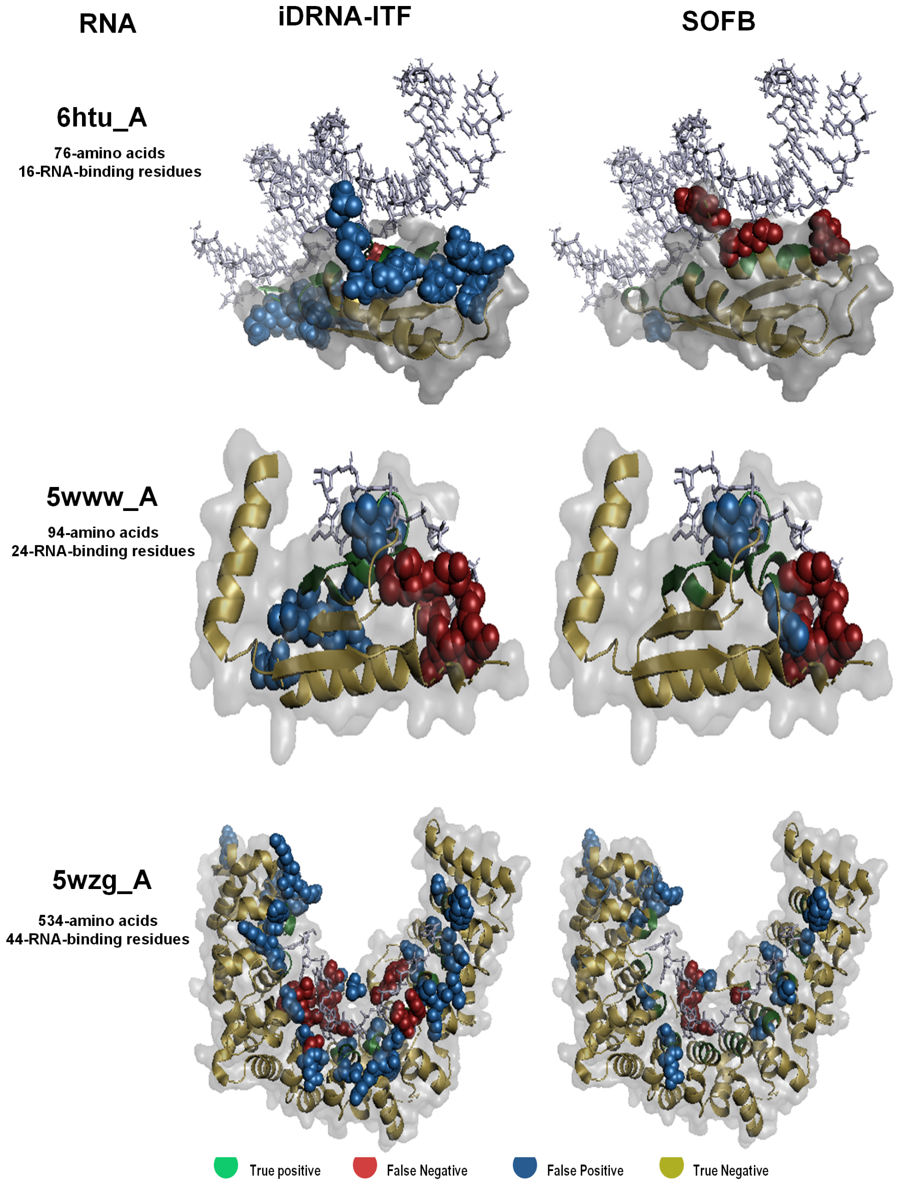

Supplementary Figure 8: Visualization of two cases predicted by SOFB and the suboptimal method iDRNA-ITF. For RNA-117_Test the top three MCC ranked proteins were selected: 6htu_A, 5www_A and 5wzg_A, showing the results of iDRNA-ITF (left) and SOFB (right) predictions. The red spheres are residues predicted to be false-negative, the blue spheres are residues predicted to be false-positive, green represents true-positive residues, and yellow represents true-negative residues. Outside the white protein surface are nucleic acids.

10 Supplementary Note 10: The details of Interpretability Analysis

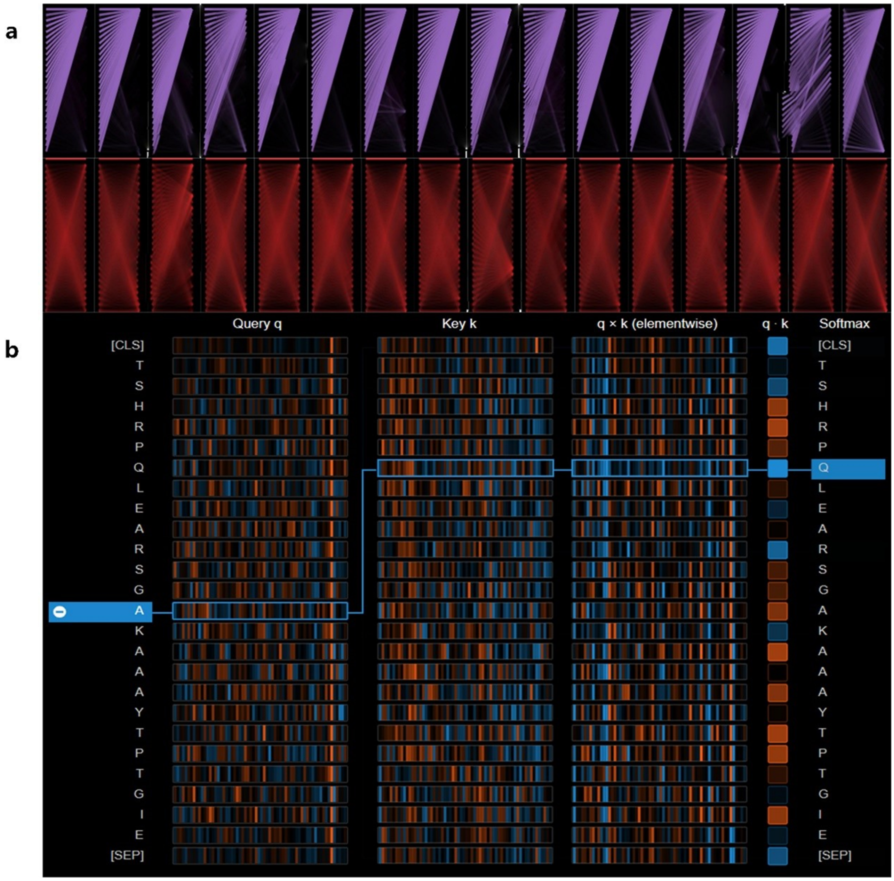

Supplementary Figure 9: (a) shows the attention heads are distributed in two different layers, with the columns representing the attention heads in each layer, and the rows representing the particular heads in each layer for RNA task. (b) shows the computation of the attention scores, where the first and second columns represent the query vector and the key vector, respectively. The vectors in the box show the two most relevant tokens in the sequence for RNA task.

We conduct additional analyses to validate our method. In particular, the two proteins with the highest MCC (two each from the DNA- and RNA-binding

test sets) were selected, and we then performed random mutations on the amino acids incorporating the positions of the binding residue. Subsequently, the mutant sequences were fed into the NABert model for calculating the attention score. Specifically, the scores of an amino acid in the 16 heads of the last layer were averaged to get the attention score for that amino acid. We subsequently conducted a statistical analysis to evaluate the differences in attention scores before and after these positional mutations. Specifically, we computed the attention scores for each mutation and performed a t-test to evaluate the significance of the differences in attention scores.

The experimental results were summarised in Supplementary Figure 10. In terms of DNA binding residue prediction, we can observe from Supplementary Figure 10 (a) that differences were observed in the attention scores before and after the mutations, with the p-value equal to or less than 0.05. For the RNA binding residue prediction, it can also be demonstrated from Supplementary Figure 10 (b) that the changes can be observed in the attention scores after the mutations. Furthermore, we observed that in both DNA binding prediction tasks and RNA binding prediction tasks, the attention scores of the mutated positions were lower compared to those before the mutations. This finding suggests that SOFB has the ability to concentrate more attention on biologically relevant positions, indicating its potential for discovering functional sites. These statistical results and hypothesis tests provide evidence for the effectiveness and potential interpretability of SOFB, offering different insights into the identification of functional sites.

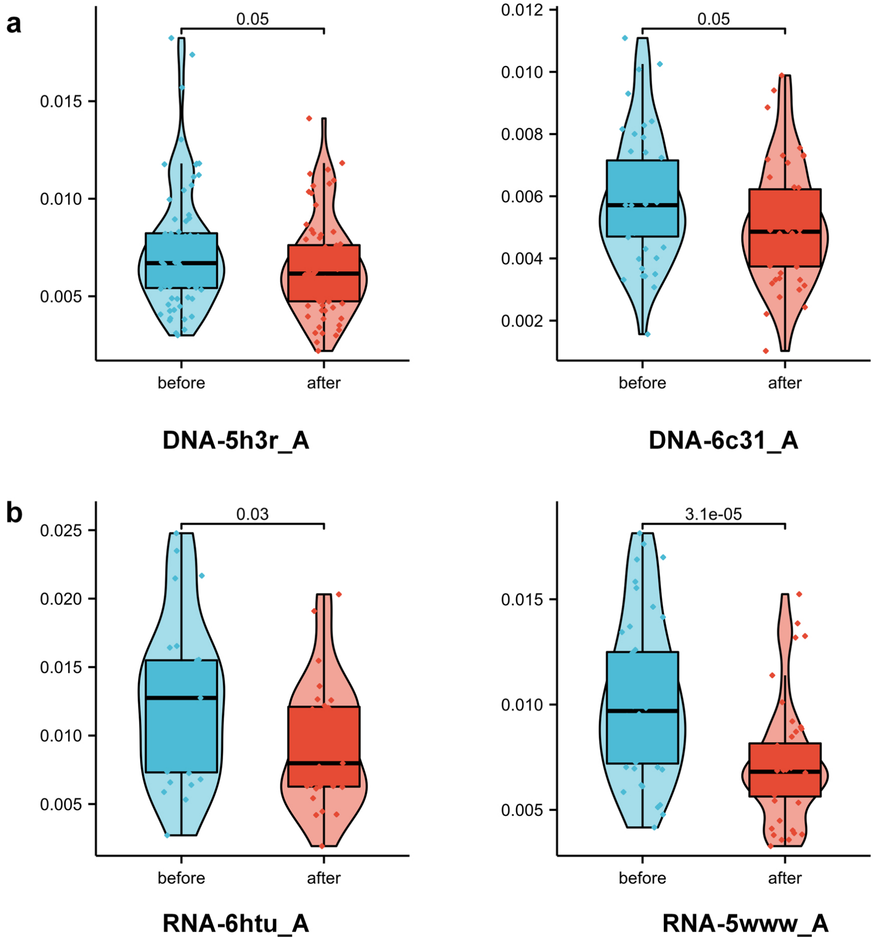

Supplementary Figure 10: shows the two T tests for SOFB on the DNA binding test set and the RNA binding test set for the two highest predicted MCC proteins, where the left side represents the scores before random substitutions and the right side represents the scores after random substitutions. p-values are shown on the top side of the graph, where the p-values before substitutions are lower than the p-values after substitutions for all the tested proteins. (For 5h3r_A, n=141; for 6c31_A, n=195; for 6htu_A, n=78; for 5www_A, n=54.)

11 Supplementary Note 11: The details of SHAP analysis

As illustrated in the Supplementary Figure 11 and Supplementary Figure 12, the first subplot on the left provides the twenty features with the highest SHAP values within the biological features, i.e., the twenty features that contributed the most to the training. The second subplot and third subplot are the analysis of the NABert embedding and the ProtT5 embedding. Each point represents a sample, where red indicates a larger feature value and blue a smaller feature value. The rightward-most points indicate higher SHAP values, which have a more positive effect on the prediction.

It can be observed that high and low values of distinct features exert both positive and negative effects on the outcome, respectively. Furthermore, features with higher SHAP values have samples that display a substantial boosting effect, which attests to the validity of most values within the features. As illustrated in the Supplementary Figure 11 (b) and Supplementary Figure 12 (b), we evaluated the performance of two features in partial residue prediction. As previously mentioned, the red color denotes a higher value for this feature and blue color indicates a lower value. Points above the axis indicates that the SHAP value is higher and has a greater effect on the prediction of the result, whereas those below the axis signify a reduced impact on prediction. Considering the twentieth sample, it demonstrates that Feature-952 possesses a higher value and a more positive effect, whereas the tenth sample indicates that Feature-339 has a lower value and a negative influence.

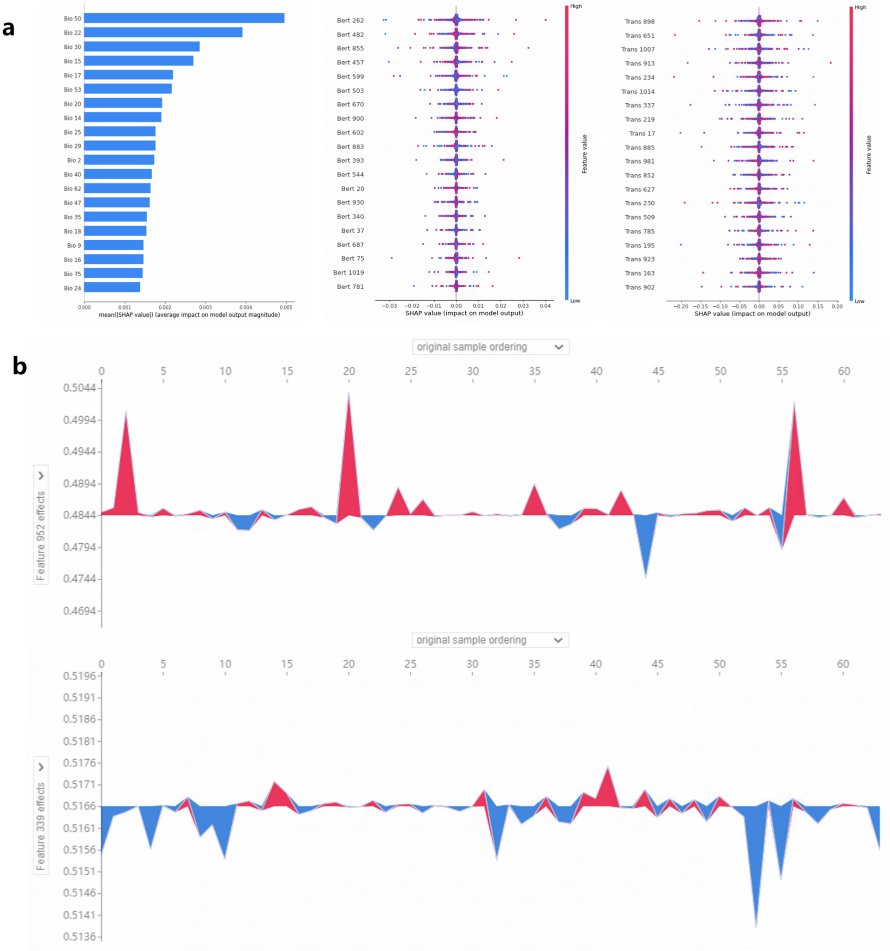

Supplementary Figure 11: (a) shows the SHAP analysis of features within SOFB in the DNA-binding residue identification task. From left to right are the features for the biological, the NABert embedding and the ProtT5 embedding, respectively; (b) shows the predicted impact of the two features on some of the samples, where the top plot visualizes the Feature-952 contribution in the ProtT5 embedding for the DNA task, and the bottom plot visualizes the Feature-339 contribution in the NABert embedding for the RNA task.

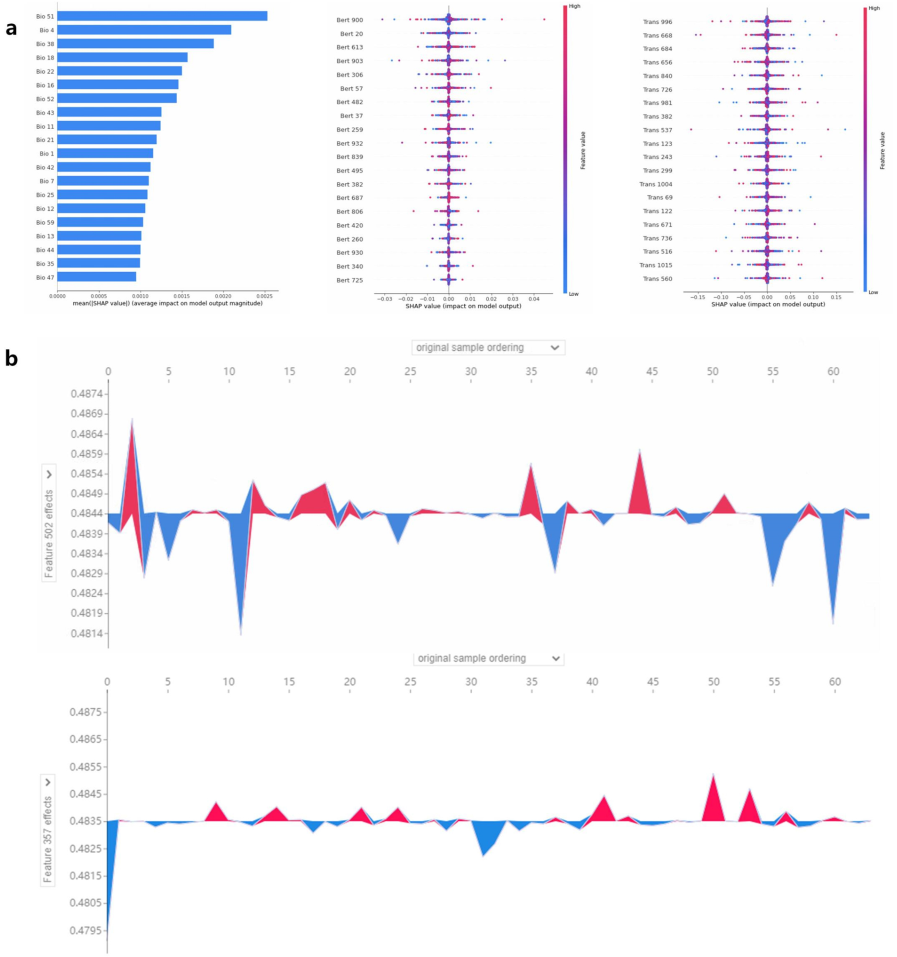

Supplementary Figure 12: (a) shows the SHAP analysis of features within SOFB in the RNA-binding residue identification task. From left to right are the features for the Biological, the NABert and the ProtT5 embedding, respectively; (b) shows the predicted impact of the two features on some of the samples, where the top plot visualizes the Feature-502 contribution in the NABert embedding for the DNA task, and the bottom visualizes the Feature-357 contribution in the ProtT5 embedding for the RNA task.

12 Supplementary Note 12: The results of SOFB that incorporate with the RoseTTAFold method

RoseTTAFoldNA [7] extends the RoseTTAFold's end-to-end deep learning approach to model the nucleic acid and protein-nucleic acid complexes, and can rapidly produces three-dimensional structure models with confidence estimates for protein-DNA and protein-RNA complexes, and for RNA tertiary structures. RoseTTAFoldNA is broadly useful for modeling the structure of naturally occurring protein-nucleic acid complexes, and for designing sequence specific RNA and DNA binding proteins.

However, it is unfortunately that the RoseTTAFoldNA model requires both protein sequences and RNA or DNA sequences to predict protein structures or DNA-protein binding, while in our study, it only involves the recognition of nucleic acid-binding residues within protein sequences, not specific nucleic acid sequences. Therefore, it is difficult to apply the RoseTTAFoldNA model into our study. Nonetheless, we conducted additional experiments and employed RoseTTAFold [8], the prototype of RoseTTAFoldNA, to generate the extensive information of protein structures and integrate it as part of the bio-information into our SOFB to predict the nucleic acid-binding residues. Specifically, after obtaining the structural information of all training and test sets, we combined them with the 75-dimensional bio-feature and obtained the 89-dimensional bio-feature for nucleic acid-binding residues prediction. Subsequently, we employed the newly integrated features to train the SOFB model, and evaluated the performance of the model on the DNA-binding and RNA-binding test set.

The experimental results are summarised in Supplementary Table 6. From the prediction results we can observe that the integration of structural information, as predicted by the advanced structural prediction model, did not yield great improvements in the predictions. Despite a decrease in the Precision (Pre), the integration of new features in SOFB (SOFB-R) demonstrated no obvious differences in other metrics such as AUC and MCC. Notably, in terms of Recall metric, SOFB-R exhibited a 1% improvement in predicting RNA binding, suggesting that the protein structural features predicted by the RoseTTAFold method have provided certain assistance in enhancing the performance of SOFB. In summary, these results demonstrate the robustness and effectiveness of the original SOFB model. Furthermore, the incorporation of the RoseTTAFold method enriches the biological features of SOFB and enhances its performance.

Supplementary Table 6: Results of SOFB on two nucleic-acid-binding test sets using structural information predicted by Rosettafold versus not using predicted structure information by Rosettafold. Source data are provided with this paper.

	Methods	Pre	Rec	F1	MCC	AUC
DNA-129_Test	SOFB-R	0.707	0.403	0.514	0.496	0.932
	SOFB	0.730	0.404	0.520	0.505	0.934
RNA-117_Test	SOFB-R	0.546	0.267	0.358	0.331	0.860
	SOFB	0.585	0.258	0.358	0.337	0.865

Supplementary References

[1] Rives, A., Meier, J., Sercu, T., Goyal, S., Lin, Z., Liu, J., Guo, D., Ott, M., Zitnick, C.L., Ma, J., *et al.*: Biological structure and function emerge from scaling unsupervised learning to 250 million protein sequences. Proceedings of the National Academy of Sciences **118**(15), 2016239118 (2021)

[2] Lin, Z., Akin, H., Rao, R., Hie, B., Zhu, Z., Lu, W., Smetanin, N., dos Santos Costa, A., Fazel-Zarandi, M., Sercu, T., Candido, S., et al.: Language models of protein sequences at the scale of evolution enable accurate structure prediction. bioRxiv (2022)

[3] Wang, N., Yan, K., Zhang, J., Liu, B.: idrna-itf: identifying dna-and rna-binding residues in proteins based on induction and transfer framework. Briefings in Bioinformatics **23**(4), 236 (2022)

[4] Yang, J., Roy, A., Zhang, Y.: Biolip: a semi-manually curated database for biologically relevant ligand–protein interactions. Nucleic Acids Research **41**, 1096–1103 (2012)

[5] Paysan-Lafosse, T., Blum, M., Chuguransky, S., Grego, T., Pinto, B.L., Salazar, G., Bileschi, M., Bork, P., Bridge, A., Colwell, L., Gough, J., Haft, D., Letunić, I., Marchler-Bauer, A., Mi, H., Natale, D., Orengo, C., Pandurangan, A., Rivoire, C., Sigrist, C.J.A., Sillitoe, I., Thanki, N., Thomas, P.D., Tosatto, S.C.E., Wu, C., Bateman, A.: InterPro in 2022. Nucleic Acids Research **51**(D1), 418–427 (2022) https://academic.oup.com/nar/article-pdf/51/D1/D418/48440814/gkac993.pdf. https://doi.org/10.1093/nar/gkac993

[6] Su, H., Liu, M., Sun, S., Peng, Z., Yang, J.: Improving the prediction of protein-nucleic acids binding residues via multiple sequence profiles and the consensus of complementary methods. Bioinformatics **35**, 930–936 (2018)

[7] Baek, M., McHugh, R., Anishchenko, I., Jiang, H., Baker, D., DiMaio, F.: Accurate prediction of protein–nucleic acid complexes using rosettafoldna. Nature Methods, 1–5 (2023)

[8] Baek, M., DiMaio, F., Anishchenko, I., Dauparas, J., Ovchinnikov, S., Lee, G.R., Wang, J., Cong, Q., Kinch, L.N., Schaeffer, R.D., *et al.*: Accurate prediction of protein structures and interactions using a three-track neural network. Science **373**(6557), 871–876 (2021)
