# Supplementary material for: SOFB is a comprehensive ensemble deep learning approach for elucidating and characterizing protein-nucleic-acid-binding residues
Source: Commun Biol. 2024 Jun 3;7:679. doi: 10.1038/s42003-024-06332-0 (PMC11148103; doi:10.1038/s42003-024-06332-0)
Supplement: Supplementary file 2 — Description of Additional Supplementary Files [file 42003_2024_6332_MOESM2_ESM.pdf]

Description of Additional Supplementary Files

File name: Supplementary Data
Description: The numerical source data for the graphs in the main figures.
